# Different Populations of Blacklegged Tick Nymphs Exhibit Differences in Questing Behavior That Have Implications for Human Lyme Disease Risk

**DOI:** 10.1371/journal.pone.0127450

**Published:** 2015-05-21

**Authors:** Isis M. Arsnoe, Graham J. Hickling, Howard S. Ginsberg, Richard McElreath, Jean I. Tsao

**Affiliations:** 1 Department of Fisheries and Wildlife, Michigan State University, East Lansing, Michigan, United States of America; 2 Center for Wildlife Health, University of Tennessee Institute of Agriculture, Knoxville, Tennessee, United States of America; 3 United States Geological Survey Patuxent Wildlife Research Center, Rhode Island Field Station, University of Rhode Island, Kingston, Rhode Island, United States of America; 4 Department of Anthropology and Center for Population Biology, University of California Davis, Davis, California, United States of America; University of Minnesota, UNITED STATES

## Abstract

Animal behavior can have profound effects on pathogen transmission and disease incidence. We studied the questing (= host-seeking) behavior of blacklegged tick (*Ixodes scapularis*) nymphs, which are the primary vectors of Lyme disease in the eastern United States. Lyme disease is common in northern but not in southern regions, and prior ecological studies have found that standard methods used to collect host-seeking nymphs in northern regions are unsuccessful in the south. This led us to hypothesize that there are behavior differences between northern and southern nymphs that alter how readily they are collected, and how likely they are to transmit the etiological agent of Lyme disease to humans. To examine this question, we compared the questing behavior of *I*. *scapularis* nymphs originating from one northern (Lyme disease endemic) and two southern (non-endemic) US regions at field sites in Wisconsin, Rhode Island, Tennessee, and Florida. Laboratory-raised uninfected nymphs were monitored in circular 0.2 m^2^ arenas containing wooden dowels (mimicking stems of understory vegetation) for 10 (2011) and 19 (2012) weeks. The probability of observing nymphs questing on these stems (2011), and on stems, on top of leaf litter, and on arena walls (2012) was much greater for northern than for southern origin ticks in both years and at all field sites (19.5 times greater in 2011; 3.6–11.6 times greater in 2012). Our findings suggest that southern origin *I*. *scapularis* nymphs rarely emerge from the leaf litter, and consequently are unlikely to contact passing humans. We propose that this difference in questing behavior accounts for observed geographic differences in the efficacy of the standard sampling techniques used to collect questing nymphs. These findings also support our hypothesis that very low Lyme disease incidence in southern states is, in part, a consequence of the type of host-seeking behavior exhibited by southern populations of the key Lyme disease vector.

## Introduction

The blacklegged or deer tick (*Ixodes scapularis*) vectors *Borrelia burgdorferi*, the etiological agent of Lyme disease (LD), which is the most common vector-borne disease in the United States (US) [1]. Despite the widespread presence of blacklegged ticks throughout the eastern US [2, 3], there is pronounced geographical variation in LD incidence in that region. Ninety-five percent of human LD cases in the US are reported from ‘Lyme endemic’ states in northeast and upper midwest regions [4]; LD incidence in southeast regions is over an order of magnitude lower [5]. We refer to this latitudinal variation in disease incidence in the eastern US as the “Lyme Disease Gradient”.

*Ixodes scapularis* has three parasitic life stages (larva, nymph, adult), but only the bites of nymphs and adults transmit *B*. *burgdorferi*. There is neglible transovarial transmission of the pathogen therefore larvae are considered to be born uninfected [6]. The nymphal stage is regarded as the most epidemiologically important for LD transmission because of its small size and spring/summer seasonality that coincides with human outdoor activity [7, 8]. The density of infected nymphs (DIN) is considered a useful predictor of LD risk [9–15]. Factors that influence DIN (including tick survivorship [16], host composition [17, 18], and abiotic variables [16]) are thought to influence the magnitude of risk [11, 19–21].

DIN is calculated by multiplying the density of nymphs collected in a given area by the *B*. *burgdorferi* infection prevalence of those nymphs [9, 14, 15, 22]. Ticks in such studies are typically collected by ‘dragging’ or ‘flagging’ a 1 m^2^ white cloth through the understory vegetation, as this is considered to be the most reliable and efficient method for sampling nymphal *I*. *scapularis* populations in the northeastern US [23, 24]. These methods intentionally target ticks that are questing (= host-seeking) on or above the leaf litter, as these are the ticks most likely to encounter humans. Flagging and dragging are less likely to collect ticks beneath the surface of the leaf litter; however, since such ticks are unlikely to encounter humans they presumably contribute little to LD risk. Quantification of DIN by flagging/dragging thus provides a useful index of human-nymphal encounter rates. Indeed, DIN is highly correlated with the Lyme Disease Gradient [14, 25], providing support for its use as an index of human LD risk.

Several hypotheses for the Lyme Disease Gradient have been proposed. These include geographic variation in large-scale predictors such as climatic variables [26–29], biodiversity [10, 11, 19, 20, 30], and tick genetics [31, 32], which are known and/or believed to influence the abundance of questing infected nymphs. These predictors help us understand the ultimate causes of risk variation, but for planning and executing effective intervention strategies, a mechanistic understanding of factors underlying the Lyme Disease Gradient is key.

A key mechanistic factor that needs to be considered for the LD system is vector behavior. With vector-borne diseases, it is typically assumed that risk of contact with an infected vector, and therefore risk of disease, is proportional to the abundance of infected vectors [15, 33, 34]. Behavior of both hosts and vectors influences the likelihood of encounter and thus the risk of disease, and these behaviors might differ at different sites. A mechanistic hypothesis is that nymphal questing behavior varies regionally, leading to differences in tick/human contact rates that contribute to the Lyme Disease Gradient.

At least three lines of evidence support this questing behavior hypothesis. First, drag/flag sampling efficacy differs between LD endemic and non-endemic regions. These standard methods readily collect all three tick life stages in northeastern and upper midwestern regions [3, 23, 35], whereas in southeastern regions, they collect very few nymphs even at sites where adult *I*. *scapularis* are readily flagged or dragged [3, 36–38]. The presence of abundant *I*. *scapularis* adults at these southeastern sites indicates that nymphs must also be present, and this is confirmed by observations of *I*. *scapularis* nymphs commonly attached to vertebrate hosts [36, 39–41]. Second, small mammals are the primary hosts for juveniles in northern states [30, 42], whereas lizards fill this role in the south [39–41, 43, 44]; again suggesting differences in questing behavior. Third, nymphal ticks are responsible for the majority of Lyme cases in the northeast and upper midwest [7], but are rarely recorded biting humans in the south [5, 44–47]. In combination, these observations motivated us to undertake a series of experiments in which we directly examine the questing behavior of nymphs from LD endemic and non-endemic regions.

Our first experiments aimed to: (i) quantify and compare variation in questing behavior of nymphs from two southern, non-endemic sites with nymphs from one northern, endemic site (2011), and (ii) assess whether observed behavioral differences are maintained when nymphs are translocated between regions (2012), thereby acquiring insight into the relative contributions of proximate environmental conditions and genetics to observed behavioral variation. In a future publication we will describe the generality of behavioral trends for *I*. *scapularis* nymphs collected from numerous locations throughout the northern and southern US. Here, we tested nymphs derived from mothers collected in Wisconsin (WI), South Carolina (SC), and North Carolina (NC) because these locations represent areas of low, non-endemic (SC, NC) and high, endemic (WI) LD risk in the eastern half of the US: in 2013, Lyme disease incidence was 25.2, 0.7, and 0.4 per 100,000 persons in WI, SC, and NC, respectively [4]. Likewise, the sites selected for translocation experiments in 2012 (Wisconsin (WI), Rhode Island (RI), Tennessee (TN) and Florida (FL)) are areas of high and low LD risk (incidence rates per 100,000 persons in 2013 in WI, RI, TN and FL were: 25.2, 42.2, 0.2, and 0.4, respectively [4]).

## Results

### Questing behaviors differed between ticks from Lyme endemic and non-endemic regions

We used a Bayesian analysis to predict nymph questing behavior by nymph origin while allowing the influence of individual arenas, sites, and weeks to vary. Questing behavior was defined as nymph presence on stems in 2011, or on stems, leaf litter surfaces, and arena walls in 2012. From these field data, statistical models generated predictions of the posterior distribution of questing probabilities for a given nymph origin (i.e., means and 95% highest density intervals (HDIs) that include the most credible values of the posterior distribution). In addition to the model estimates, we also report summaries of the raw data (proportions of nymphs observed each year).

#### 2011—Wisconsin nymphs were far more likely than South Carolina nymphs to be observed on stems at a field site in the northern US

These data comprise 752 observations of 16 experimental arenas placed in west central Wisconsin in 2011 over a 10-week time period (May–July). For 521 (69%) observations, no nymphs were observed on stems. In the remaining observations, up to 10 nymphs were observed questing on the stems, and ≤ 8 nymphs were visible for > 95% of non-zero observations. Nymphs tested in the arenas were the F_1_ progeny of mothers collected in Wisconsin (WI) or South Carolina (SC). We fit a model to predict questing behavior as a function of tick origin. Arena, hour of observation, and sample session were fit as random effects to account for potential variation among arenas, times of day and seasons.

**Fig 1:**
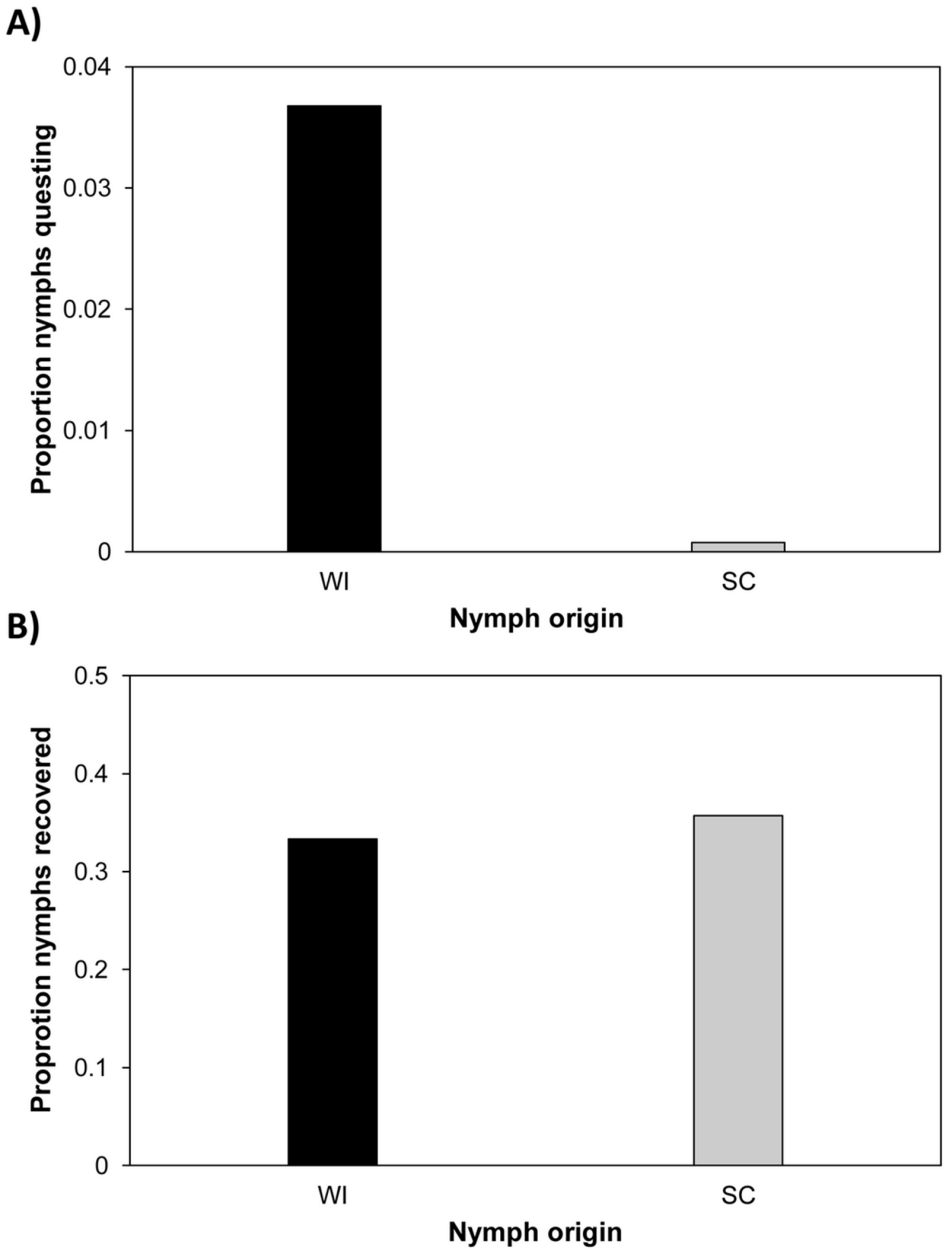
Questing behavior data from experimental arenas in Wisconsin (WI) during June-July 2011. (A) Proportion of nymphs from each origin observed questing on stems. A higher proportion of WI nymphs were observed on the stems compared to the South Carolina (SC) nymphs. (B) Proportion of nymphs from each origin recovered when arenas were depopulated on July 30, 2011. Recovery rates were similar for nymphs from both origins. The data used in this figure are given in S1 Data (A) and S2 Data (B).

The overall proportion of nymphs observed on stems in arenas in 2011 is displayed in Fig 1A; this proportion was much higher for WI nymphs (0.0368) than for SC nymphs (0.0008).

Model predictions of the probability of observing nymphs on stems as a function of nymph origin are summarized in Table 1. On average, WI nymphs were predicted to be 19.5 times as likely as SC nymphs to be observed. There was considerable variation in predicted stem use by nymphs within an origin, as reflected by the broad 95% HDI for each parameter estimate.

**Table 1:** Probability of nymph questing as a function of nymph origin.

Year	Site where tested	Posterior probability of questing by origin means (95% HDIs)			Effect size (ratio of questing)		
		WI	SC	NC	WI:SC	WI:NC	SC:NC
**2011**	WI	0.034	0.002	-	19.5*	-	-
		(0.004, 0.071)	(8.0e-9, .001)				
**2012**	FL	0.006	0.002	0.001	3.6*	7.0*	2.0
		(0.001, 0.011)	(5e-4, 0.003)	(7e-5, 2e-3)			
	TN	0.019	0.005	0.002	4.0*	11.6*	3.0
		(0.005, 0.037)	(0.001, 0.009)	(3e-4, 0.004)			
	RI	0.004	0.001	4e-4	5.0*	9.9*	2.0
		(0.001, 0.007)	(1e-4, 0.002)	(4e-5, 1e-3)			
	WI	0.020	0.005	0.002	3.9*	11.3*	2.9
		(0.005, 0.039)	(0.002, 0.009)	(3e-4, 0.004)			

Posterior distributions for models predicting probability of questing nymphs from each origin (WI, SC, NC) at each field site (WI, RI, TN and FL) in each experimental year (2011, 2012). Posterior distributions are summarized by means and 95% HDIs in parentheses. Effect sizes were calculated as the ratio of the posterior mean questing probability of one origin to another origin. In 2011, nymphs were tested only at one site (WI) and questing behavior was measured as the presence of nymphs on stems. North Carolina (NC) nymphs were not tested in 2011. In 2012, questing behavior was measured as the presence of nymphs on stems, leaf litter, and arena walls. The asterisks and bolded font indicate those comparisons for which a credible difference was evident (S1 Table). The data shown in this table are given in S1 Data (2011) and S3 Data (2012), and the R code that generated it is found in S1 Text.

To determine if the regression coefficients of the nymph origins (WI and SC) were credibly different from one another, we estimated the posterior distribution of the difference between the coefficients. Coefficients were considered to be credibly different if the HDIs of the posterior distribution of their difference did not encompass zero [48]. This analysis revealed a credible difference in the probability of observing questing WI nymphs versus questing SC nymphs (WI—SC mean = 0.032, 95% HDI = 0.004, 0.076; S1 Table).

#### 2012—Wisconsin nymphs were more likely to quest on or above the surface of the leaf litter than North Carolina or South Carolina nymphs at field sites in the northern and southern US

These data comprise 2640 observations of 66 different experimental arenas located at four sites (WI, RI, TN, and FL) in the eastern U.S. in 2012 over a 19-week time period (mid-May–mid-September). For 2059 (78%) observations, no nymphs were observed on stems, leaf litter surfaces, or the arena walls; hereafter, “questing” refers to nymphs observed in any of these locations. In the remaining observations up to 20 nymphs were observed questing, and ≤ 10 nymphs were visible for > 95% of non-zero observations. Nymphs tested in the arenas were the F_2_ progeny of mothers collected in WI or SC, or the F_1_ progeny of mothers collected in North Carolina (NC) or SC; the two generations were evenly distributed at all sites. We fit a model to predict questing behavior as a function of tick origin. Arena, site, and week were fit as random effects to account for heterogeneity among arenas, locations where nymphs were tested (sites), and time (week in which sample occurred).

**Fig 2:**
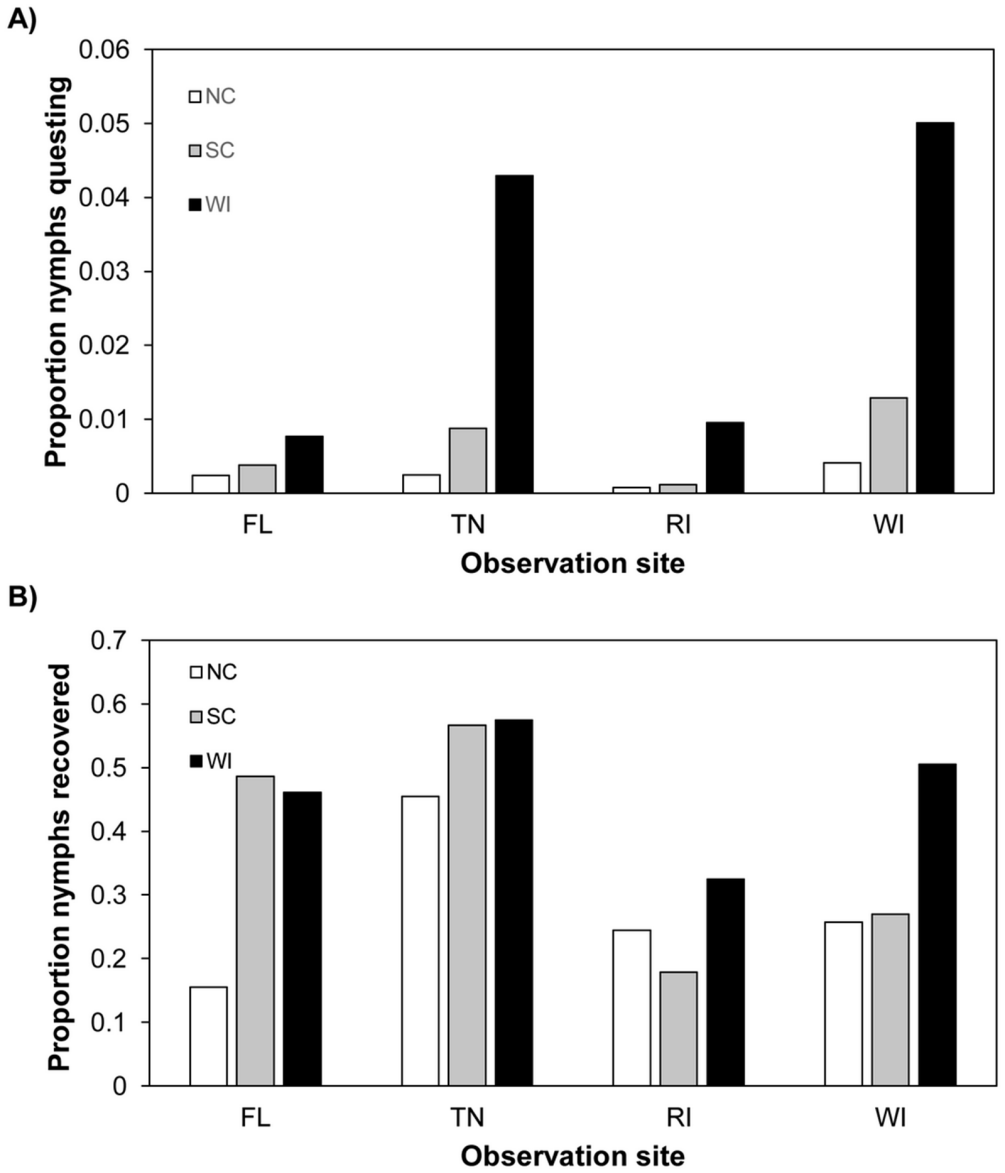
Questing behavior data from experimental arenas at northern and southern field sites; May-September, 2012. (A) Proportion of nymphs at each field site, from each origin, observed questing on stems, leaf litter, and arena walls. Wisconsin (WI) nymphs were observed in higher proportions compared to North Carolina (NC) or South Carolina (SC) nymphs at all four field sites. (B) Proportion of nymphs from each origin recovered at each field site when arenas were depopulated September 13–20, 2012. Recovery rates varied for each nymph origin at the four sites. The data used in this figure are given in (A) S3 Data and (B) S4 Data.

The overall proportion of observed questing nymphs at each site (WI, RI, TN and FL) from each origin (WI, SC and NC) in 2012 is displayed in Fig 2A. WI nymphs were observed most often at all four sites, with the total proportion questing ranging from 0.008–0.050 among the sites. Nymphs from SC were observed second most often (proportion ranging from 0.001–0.013), and NC nymphs were observed least often (proportion ranging from 0.001–0.004) at all sites.

Model predictions of the probability of observing nymphs on stems, leaf litter surfaces, or arena walls as a function of nymph origin are summarized in Table 1. On average, WI nymphs were 3.6–11.6 times as likely as those from SC or NC to be questing in arenas. As in 2011, considerable within-origin variation was observed, as reflected by the broad 95% HDIs estimates.

Comparisons involving WI nymphs (WI—SC; WI—NC) were deemed credibly different at all four sites (S1 Table). None of the SC—NC comparisons revealed credible differences (i.e. all 95% HDIs overlapped zero), indicating that the questing probabilities of these two origins were not different at any site.

The WI:SC comparison undertaken in WI in 2011 produced an effect size of 19.5 (Table 1). When this same comparison was repeated at all four observation sites in 2012, effect sizes were lower than those observed in 2011 (Table 1; range 3.6–5.0). This was due, in part, to the 'stricter' definition of questing used in 2011 (counting nymphs on stems) versus 2012 (counting nymphs on stems, leaf litter surfaces, and arena walls). When the 2011 definition was applied to the 2012 data, the effect sizes for the WI:SC comparison rose (range 8.3–14.8 for the four observation sites).

### Survival rates in 2011 did not differ among nymph origins; 2012 survival varied by origin among sites

#### Wisconsin and South Carolina nymphs showed similar survival in arenas at a field site in the northern US in 2011

The observation of greater questing on stems by WI compared to SC nymphs could have resulted from greater mortality of the southern nymphs in the northern environment. To assess this possibility, we fit a model to predict the probability of recovering nymphs from a given origin at the end of the experiment. Arena was fit as a random effect. The observed recovery of nymphs ranged from 17%- 48% of those initially released into arenas. Furthermore, the overall proportion of live nymphs recovered from arenas at the end of the experiment was similar for nymphs of both origins (WI = 0.333, SC = 0.357; Fig 1B). This similarity in recovery rates strongly suggests that the difference in questing behavior of the two groups was not a consequence of differential survival.

If nymphal questing behavior were a function of survival we would anticipate finding a positive relationship between questing behavior and recovery; visual inspection of Fig 1A and 1B reveals no such pattern. Furthermore, we did not observe a credible difference in recovery between WI and SC nymphs (WI-SC mean = -0.026, 95% HDI = -0.118, 0.080; S2 Table). Model predictions of the probability of recovering nymphs from arenas as a function of nymph origin are summarized in Table 2.

**Table 2:** Probability of nymph recovery as a function of nymph origin.

Year	Site where tested	Posterior probability of recovery by origin means (95% HDIs)			Effect size (ratio of recovery)		
		WI	SC	NC	WI:SC	WI:NC	SC:NC
**2011**	WI	0.326	0.357	-	0.9	-	-
		(0.248, 0.396)	(0.283, 0.435)				
**2012**	FL	0.442	0.392	0.146	1.1	3.0*	2.7*
		(0.270, 0.614)	(0.221, 0.574)	(0.014, 0.288)			
	TN	0.553	0.505	0.365	1.1	1.5	1.4
		(0.362, 0.735)	(0.307, 0.688)	(0.152, 0.638)			
	RI	0.385	0.195	0.210	2.0	1.8	0.9
		(0.205, 0.568)	(0.091, 0.315)	(0.065, 0.394)			
	WI	0.455	0.262	0.245	1.7*	1.9	1.1
		(0.293, 0.631)	(0.146, 0.400)	(0.076, 0.422)			

Posterior distributions for models predicting probability of recovery of nymphs from each origin (WI, SC, NC) at each field site (WI, RI, TN and FL) in each experimental year (2011, 2012). Posterior distributions are summarized by means and 95% HDIs (in parentheses). Effect sizes are calculated as the ratio of the posterior mean recovery probability of one origin to another origin. In 2011, nymphs were tested in experimental arenas for 69 days; North Carolina (NC) nymphs were not tested that year. In 2012, nymphs were tested for a much longer period (132–137 days). The asterisks and bolded font indicates those comparisons for which a credible difference was evident (S2 Table). The data shown in this table are given in S2 Data (2011) and S4 Data (2012), and the R code that generated it is found in S1 Text.

#### Wisconsin, South Carolina, and North Carolina nymphs showed varied survival at field sites in the northern and southern US in 2012

Recovery of nymphs from arenas was highly variable in 2012, ranging from 0%-91% of the number initially released. We again fit a model with the outcome variable as number of ticks recovered from an arena, and arena and site as random effects to account for heterogeneity in recovery rates among the arenas and site locations. The overall proportion of nymphs recovered from each origin varied at each site. However, the variation in recovery did not correlate with the questing patterns exhibited by nymphs of each origin at each site (Fig 2A and 2B). This suggests that differential survival was not responsible for the overall pattern of observed questing differences.

Table 2 summarizes the model predictions of the probability of recovering nymphs as a function of nymph origin at each site. WI nymphs were predicted to be 1.1–2.0 times as likely as SC nymphs, and 1.5–3.0 times as likely as NC nymphs, to be recovered at all sites (Table 2). Model parameters describing the relationships among recovery and origin were variable within and among sites, indicating that the nymphs in our study experienced a wide range of survival rates. Differences of posterior distribution of WI—SC and WI—NC recovery probabilities were not deemed credibly different at three of four sites. The SC—NC comparison was also not different at three of four sites (S2 Table). The WI origin nymphs exhibited higher survival probability than SC and NC origin nymphs at the WI site; NC origin ticks did not survive as well as WI or SC nymphs at the FL site.

Taken together, the 2011 and 2012 analyses point to a consistent behavioral pattern whereby nymphs from two southern, non-endemic origins (SC and NC) were far less likely to quest on or above the leaf litter than nymphs from one northern, endemic origin (WI). Furthermore, our survival analyses suggest these questing differences were not an artifact of differential nymph survival.

## Discussion

### Southern origin *I*. *scapularis* nymphs rarely emerge from leaf litter as compared with nymphs from a northern origin, regardless of environment

We report consistent, pronounced differences in the questing behavior of *I*. *scapularis* nymphs from southern origins (SC, NC) compared with nymphs from a northern origin (WI). The northern-derived nymphs were much more likely (3.6–19.5 times) to be observed questing above the leaf litter in experimental arenas during both years and at all sites (northern sites—WI, RI; southern sites—TN, FL) where they were observed. These results support our hypothesis that nymphs from non-endemic, southern regions typically remain in the substrate layers of the forest floor and therefore are unlikely to come into contact with human hosts. Determining the generality of this result will require further study, however additional experiments conducted with nymphs from several locations throughout the eastern US further support our hypothesis that questing differences are consistent between nymphs from northern and southern sites [49]. We suggest that this behavior of southern nymphs contributes significantly to the low incidence of human LD cases reported in the southeastern United States. Our results support the hypothesis that the Lyme Disease Gradient in the eastern US is due, in part, to regional differences in vector behavior.

Overall, the northern origin (WI) nymphs were always most likely to be seen questing above the leaf litter (Table 1). In 2011 we observed a larger relative difference in questing probability for WI nymphs (19.5 times SC nymphs) than in 2012 (3.6–5.0 times SC nymphs). Effect sizes were lower in part because of the more stringent definition of “questing” in 2011. Southern ticks will occasionally venture onto the surface of leaf litter, but they rarely climb above it. Consequently, when “questing” is defined as climbing above the litter (as we did in 2011), the difference in WI vs SC behavior is more pronounced than if questing is defined as “on or above” the litter (as in 2012). However even when corrected for this, effect sizes were still higher in 2011. That year, observers visited the arenas bi-hourly, up to 13 times during a sampling session, whereas in 2012 arenas were observed at 8-hour intervals, for no more than three times in a sampling session. We speculate that the more frequent observer visits in 2011 may have elevated the questing response of the northern nymphs but not of the southern nymphs, as the former often parasitize small mammals [30, 42] and other hosts whose cues originate from above the leaf litter, whereas the latter more typically parasitize fossorial lizards [39–41, 43, 44] whose cues originate from below the leaf litter. Our observation in 2011 that the number of nymphs seen during a sampling session (i.e. 24- or 14-hour period of recurring bi-hourly observations) was typically highest for observations in the latter half of the session supports this hypothesis. The overall lower rates of questing in 2012 are also consistent with this hypothesis. Natural variability in questing behavior could also have contributed to differences between years.

### Differences in questing cannot be explained by differences in survivorship

In 2011, we did not observe a credible difference in probability of recovery (= index of survival) for northern and southern nymphs placed in arenas for approximately 10 weeks (Table 2). In 2012, we extended the observation period, from 10 to 19 weeks, in order to explore the possibility that southern ticks might eventually quest more vigorously if given a sufficiently long opportunity to do so or if behavior varies seasonally. In nature, nymphal activity in northern regions commences in mid-spring and tapers off by mid- to late summer [3, 7, 40, 50]. Although we observed increased activity for all three nymph origins during the second half of the 2012 observation period, the northern origin ticks continued to show greater activity above the leaf litter than southern origin ticks throughout the entire observation period and at all sites (S1 Fig). Our observations of late-season questing in the arenas, but not in natural conditions [50], most likely reflect the isolation of ticks in arenas from their hosts and predators. In nature, ticks are finding hosts or being eaten, thus removing them from the system and contributing to the observed declines in questing activity over the season. The longer observation period, however, did accentuate differences in tick recovery rates among groups at each site, but these differences did not correlate with the observed questing patterns at each site. Most striking were patterns observed at the southern sites (TN and FL) where WI and SC nymphs displayed similar recovery rates, but remarkably different questing behaviors (Fig 2A and 2B). In laboratory trials WI nymphs have higher survival rates than SC nymphs when relative humidity is low (approximately 75%) [51]. Our field observations confirm this result; WI nymphs survived better than SC nymphs at the northern sites (WI and RI; Fig 2B); these had lower ambient relative humidity compared to the southern sites (S3 Table). Future studies are needed to determine if these findings reflect ecological adaptation by northern origin tick populations.

### Only a small proportion of nymphs are questing at any given time

Another key finding from this study is that on average, the probability of observing nymphs questing at a given time was very low (< 3.5% in either year; Table 1). Questing activity of *I*. *ricinus* (the LD vector in Europe) nymphs released into field plots was approximately 4–10% [52]; drag efficiency of *I*. *pacificus* (LD vector in the western US) nymphs at a field site in California was determined to be on average 5.4% [53]; and mark-recapture studies in the northeast found that drag cloths sample approximately 6% of the total *I*. *scapularis* nymphal population *at a given time* [54]. Our results suggest that these are plausible estimates for the proportions of questing nymphs from LD endemic regions, but highly overestimate the proportion of nymphs that will be dragged in non-endemic regions (Table 1). An important point is that since >90% of the nymph population will not be dragged at any given time, even small variations in questing behavior between regions with similar population densities will result in large differences in drag sampling success.

### Hypotheses for differences in questing behavior of northern and southern *I*. *scapularis* nymphs

Prior studies have demonstrated there are differences in ‘draggability’ (likelihood of being collected on drag cloths) of northern and southern nymphs [3, 23, 35–38]; the current experiment provides a mechanistic explanation for this observation. Equivalent latitudinal trends in draggability, climate, and host use are observed in *I*. *pacificus* in California, where nymphs are draggable in northern, but rarely in southern California [55]. As is the case in the eastern US, California’s LD incidence is much lower in the south of the state than in the north [55]. Below we explore non-mutually exclusive hypotheses to explain geographic differences in questing behavior.

*Ixodes scapularis* nymphs are known to show regional differences in their use of particular classes of vertebrate hosts. Northern nymphs are generally found parasitizing small mammals, particularly mice and chipmunks [30, 42]. Southern nymphs are found mainly on lizards, particularly skinks that utilize habitats below the leaf litter surface [39–41, 43, 44]. While some tick species are known to quest in vertical locations that maximize contact with their primary hosts [56, 57] it remains unclear whether *I*. *scapularis’* associations reflect host preference by nymphs, host-independent questing strategies of nymphs, differences in host ecology (abundance, distribution, behavior), and/or differences in abiotic conditions affecting the interactions between nymphs and hosts.

In general, ticks quest more often and higher when ambient air is less desiccating (i.e., high humidity and/or lower temperature; [58–60]) presumably due to *I*. *scapularis’* intolerance to dry conditions [61]. Schulze and Jordan [62] showed that *I*. *scapularis* nymphs in New Jersey were most readily collected on drag cloths in the early morning hours, when ambient relative humidity (RH) was high and temperatures were low. In 2011, we observed a similar pattern as Schulze and Jordan—nymphs were most active in the morning hours when RH was high and temps low (S2 Fig). In 2012, nymphs were most active at midnight (S3 Fig), when RH was higher, but there was little difference in the overall morning (RH similar to midnight) and evening (lower RH than midnight) activity levels. There was a slight trend toward decreased questing activity for all origins when average ambient RH was below 80% (S1 Fig); there were several weeks, however, when nymph activity remained low despite RH readings well above 80% (S1 Fig). Questing activity at the sites seemed more influenced by seasonal trends than by day-to-day variation in abiotic conditions—nymphs at all four sites exhibited higher questing activity weeks 10–19 (S1 Fig) despite fluctuating abiotic conditions. In our study, the ambient conditions at non-Lyme endemic sites (TN and FL) were typically warmer and more humid than the endemic sites (WI and RI; S3 Table). This suggests that ambient RH is unlikely to be a limiting factor for questing activity by *I*. *scapularis* ticks at the southern sites. Given that temperature and energy (lipid) consumption in ticks are positively correlated [63], the higher temperatures at the southern sites may exert a selection pressure for southern ticks to emerge less from within the leaf litter (where it is generally cooler than above the leaf litter; S3 Table). Future studies are necessary to delineate the role of temperature on questing behavior of regional tick populations. Ginsberg et al. [51] studied survival patterns of different genotypes of *I*. *scapularis* and showed reduced survival of larvae under southern versus northern conditions. There may well be a connection between this finding and ours, since reduced survival under hot conditions in the south may be a selection pressure for reduced questing above the leaf litter. However, we do not know yet whether desiccation stress was the reason for reduced survival of southern nymphs at some of the sites in 2012—this is being investigated presently.

Environment and genetic differences amongst ticks likely affect tick questing behavior. When populations display phenotypic plasticity (different phenotypes in different environments) for a trait (e.g., questing behavior), common garden experiments—where individuals with different genetic backgrounds are raised and tested together, in the same environment—can be used to determine whether the source of this variation is due to differences in genetics, environment, or an interaction of the two [64]. Genetic influences for a trait are inferred if individuals from different groups (geographic locations, in this case) are reared together in the same environment, yet continue to display phenotypic differences [64]. Our results clearly implicate a genetic component for questing behavior. We observed consistent phenotypic differences at all common garden sites (WI, RI, TN and FL) and in both years (2011 and 2012) of our experiment. In *I*. *scapularis* populations, mitochondrial lineages representing a widespread “American Clade” and more geographically limited “Southern Clade” have been identified [31], however the behavioral differences in our experiment did not correlate with nymphs’ mitochondrial type. Both WI and NC nymphs were American clade, yet WI nymphs quested substantially more often than NC nymphs. As nuclear genetic markers for *I*. *scapularis* are developed [65] it may be possible to identify markers correlating with variation in questing behavior. We were unable to directly assess the possibility of a genotype-by-environment interaction, as between observation site comparisons of questing were confounded by the necessity of employing different observers at the different sites.

## Conclusion

Our experiments demonstrate that nymphs originating from southern, non-endemic, low LD risk areas generally stayed below the leaf litter surface while nymphs from a northern, LD endemic, high-risk area were much more likely to quest on or above the leaf litter surface. This result was consistent at all sites and under markedly varying environmental conditions, which suggests that a genetic component is partially responsible for the observed variation in questing behavior.

Climatic variables [26–29], biodiversity [10, 11, 19, 30], and tick genetics [31, 32] have all been proposed as explanations for the Lyme Disease Gradient in the eastern US. Such hypotheses focus on the ultimate causes of the observed geographic variation. Here we describe questing behavior of ticks from selected northern and southern locations and demonstrate that a mechanism—differences in questing behavior—is likely contributing to the observed geographic variation in human LD risk. In a subsequent article we will address the generality of these behavior trends for nymphs collected from geographically scattered northern and southern sites [49] which will help to explain the variation in human LD risk throughout *I*. *scapularis’* distribution; this is critical information to inform the public and health workers. Future studies are needed to unravel the ultimate causes for such differences in behavior as well as how plastic and adaptable questing behavior may be. This will be especially important to help to predict disease risk in areas where blacklegged tick populations are becoming established. Specifically, as northern populations of blacklegged ticks expand southward [66–68], will the risk of Lyme disease also expand? Or, will factors currently suppressing ‘risky’ nymphal questing behavior prevail?

More broadly, this study highlights the importance of vector behavior in disease ecology. Here we provide an example of how a shift in one aspect of vector behavior can translate into profound differences in transmission of disease to humans. The impacts of vector behavior on disease transmission to humans are noted in other vector-borne disease systems including malaria [69, 70], West Nile virus encephalitis [71], and Chagas disease [72, 73]. Continued research on the role of vector behavior and disease transmission is a critical foundation upon which to build improved approaches for mitigating disease risk. In the case of LD, *I*. *scapularis* ranges continue to expand [3, 27, 35, 66, 68], and so a better understanding of regional nymph behavior will be critical for forecasting change in human risk.

## Materials and Methods

All mouse and rabbit handling and tick feeding protocols were approved through Michigan State University’s Institutional Animal Care and Use Committee (AUF # 06/09-094-00). Permission for field site use and to conduct the research was obtained from Tall Timbers Research Station and Land Conservancy, the University of Tennessee’s Forest Resources AgResearch and Education Center (FRAEC) and Fort McCoy Military Installation.

### Field Sites

In 2011, we measured the climbing behavior of nymphal *I*. *scapularis* placed at a field site at Fort McCoy Garrison, Wisconsin (latitude 44.01°N), a LD endemic area. In 2012, we expanded our study to include two LD endemic and two non-endemic sites. The 2012 LD endemic sites comprised Fort McCoy plus a site near Kingston, Rhode Island (latitude 41.48°N); the non-endemic sites were near Oak Ridge, Tennessee (latitude 36.01°N) and at Tall Timbers Research Station, Florida (latitude 30.53°N). All sites were located in mixed deciduous forests. The forest at Fort McCoy was dominated by various oaks (*Quercus* spp.), pines (*Pinus* spp.) and red maples (*Acer rubrum*), with a shrub layer of mostly tree saplings. The Rhode Island site was dominated by red maple (*A*. *rubrum*), white pine (*Pinus strobus*), and white oak (*Q*. *alba*), with tree saplings in the shrub layer. The Tennessee forest was dominated by upland oaks (*Quercus* spp.), hickory (*Carya* spp.) and yellow poplar (*Liriodendron tulipifera*), with a mixed understory containing various saplings and several invasive understory species. The Florida forest was dominated by oak (*Quercus* spp.), maple (*Acer* spp.), interspersed with shortleaf pine and a shrub layer dominated by tree saplings. Average canopy cover estimates for the sites during the study months ranged from 86%-92%. Meteorological measurements (temperature and relative humidity) were recorded hourly at each site in both years, using paired iButton data loggers (Hygrochron, Dallas Semiconductor) placed just below the surface of the leaf litter level (0 cm) and above ground (10 cm).

### Rearing Nymphs

The laboratory-reared nymphs used in 2011 originated from 22 (WI = 8, SC = 14) engorged female *I*. *scapularis* collected in November 2010 from hunter harvested deer at check stations in Monroe County, Wisconsin (latitude 44.01°N) and Aiken County, South Carolina (latitude 33.56° N). Engorged females were allowed to oviposit in individual vials in humidity chambers at 21°C and 98% relative humidity at 16:8 (L:D) hour photoperiod conditions. At 2 to 7 weeks of age, the resulting larvae were fed on female laboratory mice (ICR (CD-1) strain, *Mus musculus*) and allowed to molt into nymphs; at 2 to 4 weeks of age, these nymphs were transferred to the field sites.

Seven groups of nymphs (3 WI, 4 SC) from the 2011 colonies were used to propagate a second generation of nymphs that were used in the 2012 experiments. Two of these groups were siblings of the nymphs used in the 2011 experiments, while the remaining five groups were from other females collected at the same locations and time as the mothers of the 2011 nymphs (S4 Table). Nymphs were fed on female laboratory mice and engorged nymphs were housed individually in vials where they were allowed to molt into adults. Adults of a single origin (WI or SC) were then mated together on New Zealand White rabbits (*Oryctolagus cuniculus*) in November 2011. The resulting engorged females were maintained as described above through the ovipositional period. Resulting larvae were reared to nymphs using the 2011 protocol. In November 2011, 5 additional engorged females were collected from hunter killed deer at check stations in Hyde County, North Carolina (latitude 35.50°N) and Aiken County, South Carolina (latitude 33.56°N) to provide additional nymphs for the 2012 experiment.

### Experimental apparatus and questing observations

Ticks originating from Wisconsin (WI) and South Carolina (SC; 2011 and 2012) and North Carolina (NC; 2012) were placed at field sites in the eastern US (Wisconsin (WI) in 2011; WI, Rhode Island (RI), Tennessee (TN), and Florida (FL) in 2012). The design of the 2011 experiment consisted of 5 blocks, each containing 4 arenas. All naturally-occurring ticks were removed by heat-treating locally-obtained leaf litter before adding it to the arenas. Two arenas were not seeded with nymphs and served as experimental controls for the effectiveness of the arena barrier and leaf litter heat treatment. In 2011, there were 5 individual sightings of a single nymph (which was *not* removed when sighted) in the treated control arenas. No nymphs were recovered from the treated controls during the survival assessments. In 2012, to provide further assurance that all preexisting, local ticks were removed from the leaf litter after heat treatment and before the experimental nymphs were released, we conducted microdrags (pressing a 12 cm x 12 cm square of white flannel material against the leaf litter inside of the arenas) and carbon dioxide (CO_2_) assays (dry ice baits) in each arena at each site. In 2012, no nymphs were observed in control arenas at any of the four sites, and no nymphs were recovered from these arenas during end-of-study survival assessments.

The arena design was modified from previously published apparatuses used for measuring *Ixodes* spp. questing behavior in natural field conditions [52, 74]. Each arena consisted of a 0.5 m diameter circle of 25 cm high aluminum flashing sunk ~7 cm into the ground. A 6 cm blockade of Tree Tanglefoot Insect Barrier (Contech) was applied to the top inner rim of the arenas to prevent ticks climbing out [74]. Inside the arenas we installed 24, 3.0 mm wide bamboo dowel rods (stems), of three heights, 5 cm, 10 cm, and 20 cm. The stems were spaced in a semi-regular pattern, with the height of stem at each position randomized. These stems served to mimic understory vegetation that ticks can climb in their natural environment. Arenas were grouped in blocks of four and surrounded by a 60 cm wire mesh (2.54 cm) barrier and covered with a wire mesh lid (S4 Fig). This excluded large and medium-sized terrestrial species and birds from the arenas, while the aluminum flashing walls provided a barrier to deflect smaller terrestrial vertebrates.

**Fig 3:**
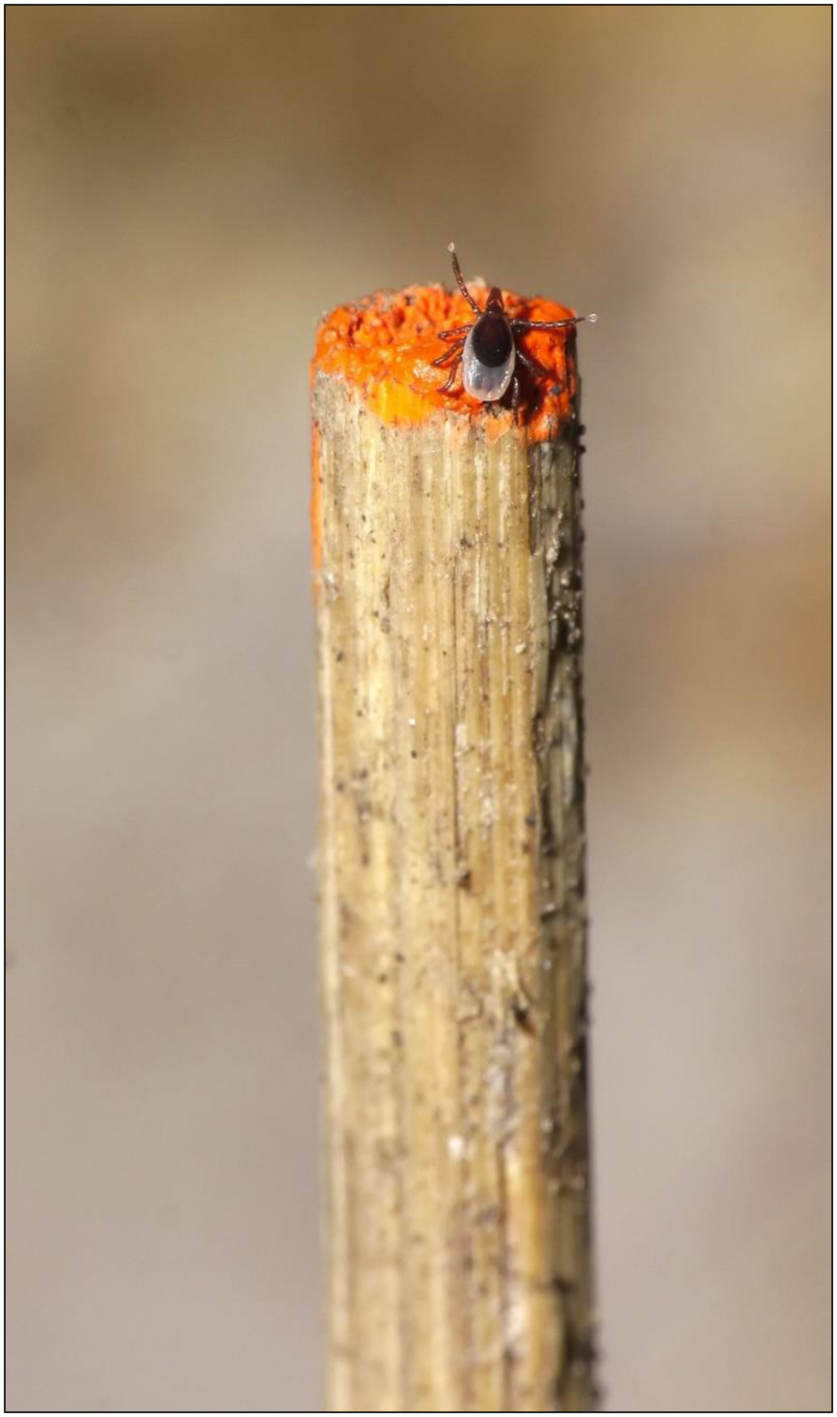
*Ixodes scapularis* nymph questing on stem in experimental arena. 1 cm of a 10 cm dowel is visible. Photo by G. Hickling.

In 2011, 16 arenas in Wisconsin each received 44–60 lab-reared nymphs of a single geographic origin (8 received WI nymphs; 8 received SC nymphs). Due to limited availability of lab-reared nymphs, some arenas contained nymphs from multiple mothers originating from the same geographic origin (S4 Table). Nymphs were deposited into the arenas on May 23, 2011. Observers, who were blind to the origin of nymphs in the arenas, recorded the number of nymphs visible on the stems during a two minute observation of the arena (Fig 3) at bi-hourly intervals during three 24-hour (June 15–16, July 7–8, July 29–30) and one 14-hour (July 5) sampling periods. Control arenas were checked in the same manner during each sampling visit. This sampling design was employed because it was not known whether *I*. *scapularis* nymphs from different origins might have divergent patterns of diel activity. The questing behavior of the SC versus WI nymphs was compared statistically based on the log-odds of their presence on stems.

In 2012, the experimental design at each site consisted of four blocks of four arenas, each arena containing 37–62 nymphal ticks from a single geographic origin (5 from WI, 3 from NC, and 8 from SC) and two unseeded additional arenas which served as controls for the leaf litter and arena barriers. The WI site had two additional arenas containing SC derived ticks. Arenas were established using the same protocol as 2011, except that the number of stems used was reduced from 24 to 15 (5 of each of the same heights used in 2011) leaving a larger area of “stem-less” leaf litter adjacent to the arena walls. Spacing between stems remained the same in both years. This design was replicated at each of the four field site locations. Nymphs were deposited into the arenas during the 1^st^ week of May, 2012. Nymph questing in arenas was recorded by observers blinded to nymph origin in the morning (approx. 0800 hours) and late afternoon (approx. 1600 hours) at weekly or biweekly intervals from May–September 2012. Additionally, a total of 18 midnight observations were carried out at 3 of the sites (FL, TN, and WI). We chose these times to conduct our observations based the periods of highest-activity of WI and SC nymphs observed in 2011 (S1 Fig). In 2012, we expanded our definition of nymph questing to include ticks on the leaf litter and arena wall, as well as on stems, after observing nymphs in these locations during the 2011 observations. We hypothesize that all such nymphs emerged from the leaf litter represent a potential risk to human hosts, not just those on stems. As ticks do not jump or fly, they must make direct contact with a host in order to attach and acquire a bloodmeal [75]. Ticks emerged from the leaf litter can instantly position their forelegs in the air to attach to a passing host. Ticks under the leaf litter (not emerged) would have difficulty making direct contact with hosts walking upon the leaf litter, as the leaf litter would create a barrier between the nymph’s forelegs and the host body. The questing behavior of the nymphs was again assessed by estimating the log-odds of observing emerged nymphs. In 2012, each site had its own set of observers.

### Tick Survival

Once behavioral observations were complete (in July 30, 2011 and mid-September 2012, respectively), we assessed the relative survival of the nymphs in each arena by conducting searches of the arena litter. In 2011, we placed air-activated hand warmers (Grabber, Byron Center, Michigan), wrapped in white flannel into the arenas for 1.5–2 hours. Nymphs attracted to the heat were removed from the flannel and placed in 95% ethanol. A 12 cm x 12 cm square of white flannel material was then pressed against the leaf litter inside of the arenas (= microdrag) and rustling the leaf litter to stir it up and expose subsurface dwelling nymphs. After the initial microdrag, a second round of microdrags was performed, again stirring the leaf litter and with moving the cloth through the leaf litter to contact in the sub-surface layers. Individuals were placed in 95% ethanol to preserve their field collected condition. In 2012, the heat pack method was abandoned because it appeared to preferentially target WI ticks (captured 53.9% of all WI ticks recovered) over SC ticks (captured 6.0% of all SC ticks recovered) and survival was assessed using only the microdragging method.

### Statistical Analyses

We used a Bayesian approach for predicting the log-odds of nymph questing behavior as a function of nymph geographic origin. No p-values are reported; but rather summaries of the posterior distributions generated from models using the data obtained. These posterior distributions describe the plausibility of possible parameter values generated from the model, given the data we observed [76]. Our goals were to quantify questing behavior and evaluate the strength of evidence for effect of geographic origin on the questing behavior of *I*. *scapularis* nymphs. We used a multilevel binomial regression model to predict the log-odds of observing questing nymphs in the arenas. Questing behavior was measured by counting the number of nymphs visible on stems (2011), or on stems, leaf litter and arena walls (2012) during a given two-minute observation of an arena. Similarly, survival was measured using a multilevel binomial regression model to predict the log-odds of recovering nymphs from arenas at the end of each study period. Survival was measured by tallying the number of nymphs recovered from arenas at the end of each experiment. We adopted a multilevel modeling approach (see Gelman and Hill [77] for an overview) for the reasons outlined by McElreath and Koster [76]; briefly, the approach simultaneously addresses our concerns regarding repeated measures and imbalanced sampling. The questing behavior models allowed for nymph questing to vary by individual arena, observation date, state of origin, and (in 2012) site of observation. The survival models allowed for nymph recovery to vary by individual arena, state of origin, and (in 2012) site of observation. Models were fitted using Stan 2.3.0 [78], a Hamiltonian Monte Carlo sampler, to draw samples from the joint posterior density of the parameters. We used weakly informative regularizing priors to analyze the data. The results we present are based on estimates derived from 3,000 samples of each parameter, after 1,000 samples for adaptation. Convergence was assessed by trace plots. To determine if the regression coefficients of the nymph origins were credibly different from one another, we estimated the posterior distribution of the difference between the coefficients. Coefficients were considered to be credibly different if the HDIs of the posterior distribution of their difference did not encompass zero [48]. Model code was generated using a convenience package for Rstan known as map2stan [79]. To visualize the results, predicted log-odds and the associated highest density interval (HDI) were back-transformed into probabilities. All statistical analyses were undertaken using R 3.1.0 (http://www.r-project.org).

## Supporting Information

S1 Data Spreadsheet containing 2011 data displayed in Fig 1, Table 1, and S1 Table. (CSV)

S2 Data Spreadsheet containing 2011 data displayed in Fig 1, Table 2, and S2 Table. (CSV)

S3 Data Spreadsheet containing 2012 data displayed in Fig 2, Table 1, and S1 Table. (CSV)

S4 Data Spreadsheet containing 2012 data displayed in Fig 2, Table 2, and S2 Table. (CSV)

S5 Data Spreadsheet containing data displayed in S1 Fig. (CSV)

S6 Data Spreadsheet containing data displayed in S2 Fig. (CSV)

S7 Data Spreadsheet containing data displayed in S3 Fig. (CSV)

S8 Data Spreadsheet containing data displayed in S3 Table. (CSV)

S1 Fig Summary of nymph questing activity and abiotic conditions at each site in 2012. Mean proportion of questing nymphs (by nymph origin-WI, SC, NC) observed in arenas during weekly observations (bar graphs, primary y-axis) at the 4 field sites in 2012: (A) Florida, (B) Rhode Island, (C) Tennessee and (D) Wisconsin. Mean ambient (10 cm) temperature and relative humidity readings for each observation week are expressed by line graphs with values on the secondary y-axis. The first column (panels A and C) shows data for the southern, non-endemic sites (FL and TN), the second column (panel B and D) shows data for the northern, endemic sites (RI and WI). NOTE: Primary y-axis differs for top and bottom rows. Although we did observe increased activity for all three nymph origins during the second half of the 2012 observation period, on average, WI nymphs (black bars) quested at higher proportions than SC and NC nymphs (grey and white bars) throughout the entire observation period and at all sites. The data used in this figure are given in S6 Data.
(TIF)

S2 Fig Bi-hourly questing activity patterns at WI field site in 2011. Proportion of nymphal *I*. *scapularis* (means ± 95CIs) of northern (WI) and southern (SC) U.S. origin, questing on stems during each observation time, in outdoor arenas in Wisconsin, June-July 2011. For both groups, emergence was highest before 0830 hours and dropped steadily with the exception of a small peak observed in the late afternoon (1230–1630 hours). The data used in this figure are given in S7 Data.
(TIF)

S3 Fig Questing activity patterns (all sites) by observation time, 2012. Proportion of nymphal *I*. *scapularis* (means ± 95 CIs) of northern (WI) and southern (NC, SC) origin, observed questing at all four sites during each observation hour (am = ~0800 hours, pm = ~1600 hours, mid = ~0000 hours), in outdoor arenas at 4 sites (WI, RI, TN, FL) in 2012. Questing was highest during the midnight observations; am and pm observations yielded similar numbers of ticks for all 3 origins. The data used in this figure are given in S8 Data.
(TIF)

S4 Fig Experimental arenas for tick questing behavior experiments 2011, 2012. Arenas were grouped in blocks of four and surrounded with wire mesh.
(TIF)

S1 Table Determination of nymph questing differences. Posterior mean difference in predicted probability of questing between origins for each site where nymphs were observed. The asterisks and bolded font indicates those comparisons for which a credible difference (HDIs do not include zero) has been determined. The data shown in this table are given in S1 Data (2011) and S3 Data (2012), and the R code that generated it is found in S1 Text.
(DOCX)

S2 Table Determination of nymph recovery differences. Posterior mean difference in predicted probability of recovery between origins for each site where nymphs were observed. The asterisks and bolded font indicates those comparisons for which a credible difference (HDIs do not include zero) has been determined. The data shown in this table are given in S2 Data (2011) and S4 Data (2012), and the R code that generated it is found in S1 Text.
(DOCX)

S3 Table Environmental conditions at field sites in 2011 and 2012. Temperature and relative humidity (means ± SD) at leaf litter (level “0 cm”) or above leaf litter (level “10 cm” = ambient) inside arenas at each field site in 2011 and 2012. Fort McCoy, Wisconsin was the only study site in 2011. In 2012, readings were discontinued after the second week of September. Latitude coordinates obtained from nationalatlas.gov. The data shown in this table are given in S5 Data.
(DOCX)

S4 Table Nymph ancestry and experimental design 2011, 2012. Number of clutches (mothers) used to propagate nymphs for behavior experiments in 2011 and 2012. Engorged females were collected from hunter harvested deer in fall of 2010 in Wisconsin (WI_2010_) and South Carolina (SC_2010_) and produced nymphs for 2011 experiments. Nymphs for the 2012 experiments were offspring of the nymphal cohort raised from the females collected in 2010. Two of 2012 clutches (WI_F2,2010*_) were directly related to the WI clutches used in 2011 arenas, while the remaining 5 clutches (WI_F2,2010_, SC_F2,2010_) were derived from mothers collected at the same time (but not related to) as those who provided clutches for 2011 arenas. Additional engorged females were collected from deer in North Carolina (NC_2011_) and South Carolina (SC_2011_) in fall of 2011 and were used to supplement the 2012 nymph supply. A single arena always contained nymphs from the same geographic origin (WI, SC or NC), however nymphs within an arena could have all been siblings from a single clutch (homogeneous) or a mixture of siblings and non-siblings from multiple clutches (heterogeneous).
(DOCX)

S1 Text Commented R code used to generate Table 1 and 2, S1 and S2 Table. (DOCX)

## References

[1] Bacon RM, Kugeler KJ, Mead PS. Surveillance for Lyme disease—United States, 1992–2006. MMWR Surveill Summ 2008 2008;57(10): 1–9. 18830214

[2] Dennis DT, Nekomoto TS, Victor JC, Paul WS, Piesman J. Reported distribution of *Ixodes scapularis* and *Ixodes pacificus* (Acari: Ixodidae) in the United States. J Med Entomol. 1998;35(5): 629–638. 9775584 10.1093/jmedent/35.5.629

[3] Diuk-Wasser MA, Gatewood AG, Cortinas MR, Yaremych-Hamer S, Tsao J, Kitron U, et al Spatiotemporal patterns of host-seeking *Ixodes scapularis* nymphs (Acari: Ixodidae) in the United States. J Med Entomol. 2006;43(2): 166–176. 16619595 10.1603/0022-2585(2006)043[0166:spohis]2.0.co;2

[4] Centers for Disease Control and Prevention. Lyme disease data-Statistics. [updated 2014 Aug 27]. Available from: http://www.cdc.gov/lyme/stats/index.html. Accessed 2014 Sep 3.

[5] Stromdahl EY, Hickling GJ. Beyond lyme: Aetiology of tick-borne human diseases with emphasis on the south-eastern United States. Zoonoses and Public Health. 2012;59: 48–64. 10.1111/j.1863-2378.2012.01475.x 22958250

[6] Rollend L, Fish D, Childs JE. Transovarial transmission of *Borrelia* spirochetes by *Ixodes scapularis*: A summary of the literature and recent observations. Ticks Tick Borne Dis. 2013;4(1–2): 46–51. 23238242 10.1016/j.ttbdis.2012.06.008

[7] Falco RC, McKenna DF, Daniels TJ, Nadelman RB, Nowakowski J, Fish D, et al Temporal relation between *Ixodes scapularis* abundance and aisk for Lyme disease associated with erythema migrans. Am J Epidemiol. 1999;149(8): 771–776. 10206627 10.1093/oxfordjournals.aje.a009886

[8] Barbour A, Fish D. The biological and social phenomenon of Lyme disease. Science. 1993;260(5114): 1610–1616. 8503006 10.1126/science.8503006

[9] Ostfeld RS, Keesing F. Biodiversity series: The function of biodiversity in the ecology of vector-borne zoonotic diseases. Can J Zool. 2000;78(12): 2061–2078.

[10] Schmidt KA, Ostfeld RS. Biodiversity and the dilution effect in disease ecology. Ecology. 2001;82(3): 609–619.

[11] Allan BF, Keesing F, Ostfeld RS. Effect of forest fragmentation on Lyme disease risk. Conserv Biol. 2003;17(1): 267–272.

[12] Horobik V, Keesing F, Ostfeld R. Abundance and *Borrelia burgdorferi*-infection prevalence of nymphal *Ixodes scapularis* ticks along forest—field edges. EcoHealth. 2006;3(4): 262–268.

[13] Hoen AG, Margos G, Bent SJ, Diuk-Wasser MA, Barbour A, Kurtenbach K, et al Phylogeography of *Borrelia burgdorferi* in the eastern United States reflects multiple independent Lyme disease emergence events. Proc Natl Acad Sci U S A. 2009;106(35): 15013–15018. 10.1073/pnas.0903810106 19706476 PMC2727481

[14] Diuk-Wasser MA, Hoen AG, Cislo P, Brinkerhoff R, Hamer SA, Rowland M, et al Human risk of infection with *Borrelia burgdorferi*, the Lyme disease agent, in eastern United States. Am J Trop Med Hyg. 2012;86(2): 320–327. 10.4269/ajtmh.2012.11-0395 22302869 PMC3269287

[15] Mather TN, Nicholson MC, Donnelly EF, Matyas BT. Entomologic index for human risk of Lyme disease. Am J Epidemiol. 1996;144(11): 1066–1069. 8942438 10.1093/oxfordjournals.aje.a008879

[16] Berger KA, Ginsberg HS, Gonzalez L, Mather TN. Relative humidity and activity patterns of *Ixodes scapularis* (Acari: Ixodidae). J Med Entomol. 2014;51(4): 769–776. 25118408 10.1603/me13186

[17] Swei A, Ostfeld RS, Lane RS, Briggs CJ. Impact of the experimental removal of lizards on Lyme disease risk. Proc R Soc B. 2011;278: 2970–2978. 10.1098/rspb.2010.2402 21325326 PMC3151702

[18] Bouchard C, Beauchamp G, Leighton PA, Lindsay R, Bélanger D, Ogden NH. Does high biodiversity reduce the risk of Lyme disease invasion. Parasit Vectors. 2013;6(1): 195. 23816142 10.1186/1756-3305-6-195 PMC3728044

[19] Ostfeld RS, Keesing F. Biodiversity and disease risk: The case of Lyme disease. Conserv Biol. 2000;14(3): 722–728.

[20] Keesing F, Holt RD, Ostfeld RS. Effects of species diversity on disease risk. Ecol Lett. 2006;9(4): 485–498. 16623733 10.1111/j.1461-0248.2006.00885.x

[21] Ogden NH, Tsao JI. Biodiversity and Lyme disease: Dilution or amplification? Epidemics. 2009;1(3): 196–206. 10.1016/j.epidem.2009.06.002 21352766

[22] Stafford KC, Cartter ML, Magnarelli LA, Ertel S-H, Mshar PA. Temporal correlations between tick abundance and prevalence of ticks infected with *Borrelia burgdorferi* and increasing incidence of Lyme disease. J Clin Microbiol. 1998;36(5): 1240–1244. 9574684 10.1128/jcm.36.5.1240-1244.1998 PMC104807

[23] Falco R, Fish D. A comparison of methods for sampling the deer tick, *Ixodes dammini*, in a Lyme disease endemic area. Exp Appl Acarol. 1992;14(2): 165–173. 1638929 10.1007/BF01219108

[24] Ginsberg H, Ewing C. Comparison of flagging, walking, trapping, and collecting from hosts as sampling methods for northern deer ticks, *Ixodes dammini*, and lone-star ticks, *Amblyomma americanum* (Acari: Ixodidae). Exp Appl Acarol. 1989;7(4): 313–322. 2806016 10.1007/BF01197925

[25] Pepin KM, Eisen RJ, Mead PS, Piesman J, Fish D, Hoen AG, et al Geographic variation in the relationship between human Lyme disease incidence and density of infected host-seeking *Ixodes scapularis* nymphs in the eastern United States. Am J Trop Med Hyg. 2012;86(6): 1062–1071. 10.4269/ajtmh.2012.11-0630 22665620 PMC3366524

[26] Brownstein JS, Holford TR, Fish D. Effect of climate change on Lyme disease risk in North America. EcoHealth. 2005;2(1): 38–46. 19008966 10.1007/s10393-004-0139-x PMC2582486

[27] Ogden NH, St-Onge L, Barker IK, Brazeau S, Bigras-Poulin M, Charron DF, et al Risk maps for range expansion of the Lyme disease vector, *Ixodes scapularis*, in Canada now and with climate change. Int J Health Geogr. 2008; 10.1186/1476-072X-7-24 PMC2412857 18498647

[28] Ogden NH, Radojevic M, Wu X, Duvvuri VR, Leighton PA, Wu J. Estimated effects of projected climate change on the basic reproductive number of the Lyme disease vector *Ixodes scapularis* . Environ Health Perspect. 2014;122(6): 631–638. 10.1289/ehp.1307799 24627295 PMC4050516

[29] Feria-Arroyo T, Castro-Arellano I, Gordillo-Perez G, Cavazos A, Vargas-Sandoval M, Grover A, et al Implications of climate change on the distribution of the tick vector *Ixodes scapularis* and risk for Lyme disease in the Texas-Mexico transboundary region. Parasites & Vectors. 2014;7(1): 199. 24766735 10.1186/1756-3305-7-199 PMC4022269

[30] LoGiudice K, Ostfeld RS, Schmidt KA, Keesing F. The ecology of infectious disease: Effects of host diversity and community composition on Lyme disease risk. Proc Natl Acad Sci U S A. 2003;100(2): 567–571. 12525705 10.1073/pnas.0233733100 PMC141036

[31] Qiu WG, Dykhuizen DE, Acosta MS, Luft BJ. Geographic uniformity of the Lyme disease spirochete (*Borrelia burgdorferi*) and its shared history with tick vector (*Ixodes scapularis*) in the northeastern United States. Genetics. 2002;160: 833–849. 11901105 10.1093/genetics/160.3.833 PMC1462027

[32] Humphrey PT, Caporale DA, Brisson D. Uncoordinated phylogeograph of *Borrelia burgdorferi* and its tick vector, *Ixodes scapularis* . Evolution. 2010;64(9): 2653–2663. 10.1111/j.1558-5646.2010.01001.x 20394659 PMC2919648

[33] Moore C, McLea RG, Mitchell CJ, Nasci RS, Tsai TF, Calisher C, et al Guidlines for arbovirus surveillance programs in the United States. Washington, DC; 1993.

[34] Mills JN, Gage KL, Khan AS. Potential influence of climate change on vector-borne and zoonotic diseases: A review and proposed research plan. Environ Health Perspect. 2010;118(11): 1507–1514. 10.1289/ehp.0901389 20576580 PMC2974686

[35] Hamer S, Tsao J, Walker E, Hickling G. Invasion of the Lyme disease vector *Ixodes scapularis*: Implications for *Borrelia burgdorferi* endemicity. EcoHealth. 2010;7(1): 47–63. 10.1007/s10393-010-0287-0 20229127

[36] Rogers AJ. A study of the ixodid ticks of northern Florida, including the biology of life history of *Ixodes scapularis* say (Ixodidae: Acarina). PhD dissertation, University of Maryland. 1953.

[37] Goltz L, Goddard J. Observations on the seasonality of *Ixodes scapularis* say in Mississippi, USA. Syst Appl Acarol. 2013;18(3): 212–217.

[38] Goddard J, Piesman J. New records of immature *Ixodes scapularis* from Mississippi. J Vector Ecol. 2006;31(2): 421–422. 17249363 10.3376/1081-1710(2006)31[421:nroiis]2.0.co;2

[39] Apperson CS, Levine JF, Evans TL, Braswell A, Heller J. Relative utilization of reptiles and rodents as hosts by immature *Ixodes scapularis* (Acari, Ixodidae) in the coastal-plain of North Carolina, USA. Exp Appl Acarol. 1993;17(10): 719–731. 7628223 10.1007/BF00051830

[40] Kollars TM, Oliver JH, Kollars PG, Durden LA. Seasonal activity and host associations of *Ixodes scapularis* Acari: Ixodidae) in southeastern Missouri. J Med Entomol. 1999;36(6): 720–726. 10593072 10.1093/jmedent/36.6.720

[41] Durden L, Oliver J, Banks C, Vogel G. Parasitism of lizards by immature stages of the blacklegged tick, *Ixodes scapularis* (Acari, Ixodidae). Exp Appl Acarol. 2002;26(3–4): 257–266. 12537298 10.1023/a:1021199914816

[42] Spielman A, Wilson ML, Levine JF, Piesman J. Ecology of *Ixodes dammini*-borne human babesiosis and lyme disease. Annu Rev Entomol. 1985;30(1): 439–460. 3882050 10.1146/annurev.en.30.010185.002255

[43] Levine JF, Apperson CS, Howard P, Washburn M, Braswell AL. Lizards as hosts for immature *Ixodes scapularis* (Acari: Ixodidae) in North Carolina. J Med Entomol. 1997;34(6): 594–598. 9439111 10.1093/jmedent/34.6.594

[44] Oliver JH. Lyme borreliosis in the southern United States: A review. J Parasitol. 1996;82(6): 926–935. 8973401

[45] Felz MW, Durden LA, Oliver JH Jr. Ticks parasitizing humans in Georgia and South Carolina. J Parasitol. 1996;82(3): 505–508. 8636862

[46] Goddard J. A ten-year study of tick biting in Mississippi: Implications for human disease transmission. J Agromedicine. 2002;8(2): 25–32. 12853269 10.1300/J096v08n02_06

[47] Williamson PC, Billingsley PM, Teltow GJ, Seals JP, Turnbough MA, Atkinson SF. *Borrelia*, *ehrlichia*, and *rickettsia* spp. In ticks removed from persons, Texas, USA. Emerg Infect Dis. 2010;16(3): 441–446. 10.3201/eid1603.091333 20202419 PMC3322032

[48] Kruschke JK, Aguinis H, Joo H. The time has come: Bayesian methods for data analysis in the organizational sciences. Organ Res Methods. 2012;15(4): 722–752.

[49] Arsnoe IM. Variation in blacklegged tick *Ixodes scapularis* questing behavior has implications for human Lyme disease risk in the eastern United States. PhD dissertation, Michigan State University; 2015.

[50] Gatewood AG, Liebman KA, Vourc'h G, Bunikis J, Hamer SA, Cortinas R, et al Climate and tick seasonality are predictors of borrelia burgdorferi genotype distribution. Appl Environ Microbiol. 2009;75(8): 2476–2483. 10.1128/AEM.02633-08 19251900 PMC2675205

[51] Ginsberg H, Rulison E, Azevedo A, Pang G, Kuczaj I, Tsao J, et al Comparison of survival patterns of northern and southern genotypes of the North American tick *Ixodes scapularis* (Acari: Ixodidae) under northern and southern conditions. Parasit Vectors. 2014;7(1): 394. 25160464 10.1186/1756-3305-7-394 PMC4153913

[52] Dautel H, Dippel C, Kämmer D, Werkhausen A, Kahl O. Winter activity of *Ixodes ricinus* in a Berlin forest. Int J Med Microbiol. 2008;298: 50–54.

[53] Tälleklint-Eisen L, Lane RS. Efficiency of drag sampling for estimating population sizes of *Ixodes pacificus* (Acari: Ixodidae) nymphs in leaf litter. J Med Entomol. 2000;37(3): 484–487. 15535598 10.1093/jmedent/37.3.484

[54] Daniels TJ, Falco RC, Fish D. Estimating population size and drag sampling efficiency for the blacklegged tick (Acari: Ixodidae). J Med Entomol. 2000;37(3): 357–363. 15535578 10.1603/0022-2585(2000)037[0357:EPSADS]2.0.CO;2

[55] Lane RS, Fedorova N, Kleinjan JE, Maxwell M. Eco-epidemiological factors contributing to the low risk of human exposure to ixodid tick-borne borreliae in southern California, USA. Ticks Tick Borne Dis. 2013;4(5): 377–385. 10.1016/j.ttbdis.2013.02.005 23643357

[56] Mejlon HA, Jaenson TGT. Questing behaviour of *Ixodes ricinus* ticks (Acari: Ixodidae). Exp Appl Acarol. 1997;21(12): 747–754. 10.1023/a:1018473122070 9423270

[57] Tsunoda T, Tatsuzawa S. Questing height of nymphs of the bush tick, *Haemaphysalis longicornis*, and its closely related species, *H*. *mageshimaensis*: Correlation with body size of the host. Parasitology. 2004;128(05): 503–509. 15180318 10.1017/s0031182004004913

[58] Randolph SE, Storey K. Impact of microclimate on immature tick-rodent host interactions (Acari: Ixodidae): Implications for parasite transmission. J Med Entomol. 1999;36(6): 741–748. 10593075 10.1093/jmedent/36.6.741

[59] Vail SG, Smith G. Vertical movement and posture of blacklegged tick (Acari: Ixodidae) nymphs as a function of temperature and relative humidity in laboratory experiments. J Med Entomol. 2002;39(6): 842–846. 12495181 10.1603/0022-2585-39.6.842

[60] Vail SG, Smith G. Air temperature and relative humidity effects on behavioral activity of blacklegged tick (Acari: Ixodidae) nymphs in New Jersey. J Med Entomol. 1998;35(6): 1025–1028. 9835697 10.1093/jmedent/35.6.1025

[61] Needham GR, Teel PD. Off-host physiological ecology of ixodid ticks. Annu Rev Entomol. 1991;36(1): 659–681. 2006871 10.1146/annurev.en.36.010191.003303

[62] Schulze TL, Jordan RA. Meteorologically mediated diurnal questing of *Ixodes scapularis* and *Amblyomma americanum* (Acari: Ixodidae) nymphs. J Med Entomol. 2003;40(4): 395–402. 14680102 10.1603/0022-2585-40.4.395

[63] van Es RP, Hillerton JE, Gettinby G. Lipid consumption in *Ixodes ricinus* (Acari: Ixodidae): Temperature and potential longevity. Bull Entomol Res. 1998;88(05): 567–573.

[64] Conner JK, Hartl DL. A primer of ecological genetics. Sinauer Associates Incorporated; 2004.

[65] Van Zee J, Black WC, Levin M, Goddard J, Smith J, Piesman J. High snp density in the blacklegged tick, *Ixodes scapularis*, the principal vector of Lyme disease spirochetes. Ticks Tick Borne Dis. 2013;4(1–2): 63–71. 23219364 10.1016/j.ttbdis.2012.07.005

[66] Cortinas MR, Kitron U. County-level surveillance of white-tailed deer infestation by *Ixodes scapularis* and *Dermacentor albipictus* (Acari: Ixodidae) along the Illinois river. J Med Entomol. 2006;43(5): 810–819. 17017213 10.1603/0022-2585(2006)43[810:csowdi]2.0.co;2

[67] Kelly RR, Gaines D, Gilliam WF, Brinkerhoff RJ. Population genetic structure of the Lyme disease vector *Ixodes scapularis* at an apparent spatial expansion front. Infect Genet Evol. 2014;27(0): 543–550. 10.1016/j.meegid.2014.05.022 24882702

[68] Wang P, Glowacki M, Hoet AE, Needham GR, Smith KA, Gary RE, et al Emergence of *Ixodes scapularis* and *Borrelia burgdorferi*, the Lyme disease vector and agent, in Ohio. Front Cell Infect Microbiol. 2014;4. 10.3389/fcimb.2014.00070 PMC4044495 24926441

[69] Day JF. Host-seeking strategies of mosquito disease vectors. J Am Mosq Control Assoc. 2005;21(sp1): 17–22. 16921679 10.2987/8756-971X(2005)21[17:HSOMDV]2.0.CO;2

[70] Mahande A, Mosha F, Mahande J, Kweka E. Feeding and resting behaviour of malaria vector, *Anopheles arabiensis* with reference to zooprophylaxis. Malar J. 2007;6(100): 10.1186. 17663787 10.1186/1475-2875-6-100 PMC1964787

[71] Kilpatrick AM, Kramer LD, Jones MJ, Marra PP, Daszak P. West Nile virus epidemics in North America are driven by shifts in mosquito feeding behavior. PLoS Biol. 2006;4(4): 606–610. 10.1371/journal.pbio.0040082 PMC1382011 16494532

[72] Canals M, Solís R, Tapia C, Ehrenfeld M, Cattan P. Comparison of some behavioral and physiological feeding parameters of *Triatoma infestans* Klug, 1834 and *Mepraia spinolai* Porter, 1934, vectors of Chagas disease in Chile. Mem Inst Oswaldo Cruz. 1999;94: 687–692. 10464419 10.1590/s0074-02761999000500025

[73] Martínez-Ibarra JA, Miguel-Alvarez A, Arredondo-Jiménez JI, Rodríguez-López MH. Update on the biology of *Triatoma dimidiata* Latreille (Hemiptera: Reduviidae) under laboratory conditions. J Am Mosq Control Assoc. 2001;17(3): 209–210. 14529090

[74] Lane RS, Kleinjan JE, Schoeler GB. Diel activity of nymphal *Dermacentor occidentalis* and *Ixodes pacificus* (Acari: Ixodidae) in relation to meteorological factors and host activity periods. J Med Entomol. 1995;32(3): 290–299. 7616519 10.1093/jmedent/32.3.290

[75] Sonenshine D. Biology of ticks, vol. 1 and 2 New York: Oxford University Press; 1991.

[76] McElreath R, Koster J. Using multilevel models to estimate variation in foraging returns. Hum Nat. 2014;25(1): 100–120. 10.1007/s12110-014-9193-4 24522975

[77] Gelman A, Hill J. Data analysis using regression and multilevel/hierarchical models. New York, NY: Cambridge University Press; 2007.

[78] Stan Development Team. Stan: A C++ library for probability and sampling, version 2.3.0. R package; 2014.

[79] McElreath R. Rethinking: Statistical rethinking book package. version 1.393. R package; 2014.

