# Supplementary figures and images for: Different Populations of Blacklegged Tick Nymphs Exhibit Differences in Questing Behavior That Have Implications for Human Lyme Disease Risk
Source: PLoS One. 2015 May 21;10(5):e0127450. doi: 10.1371/journal.pone.0127450 (PMC4440738; doi:10.1371/journal.pone.0127450)

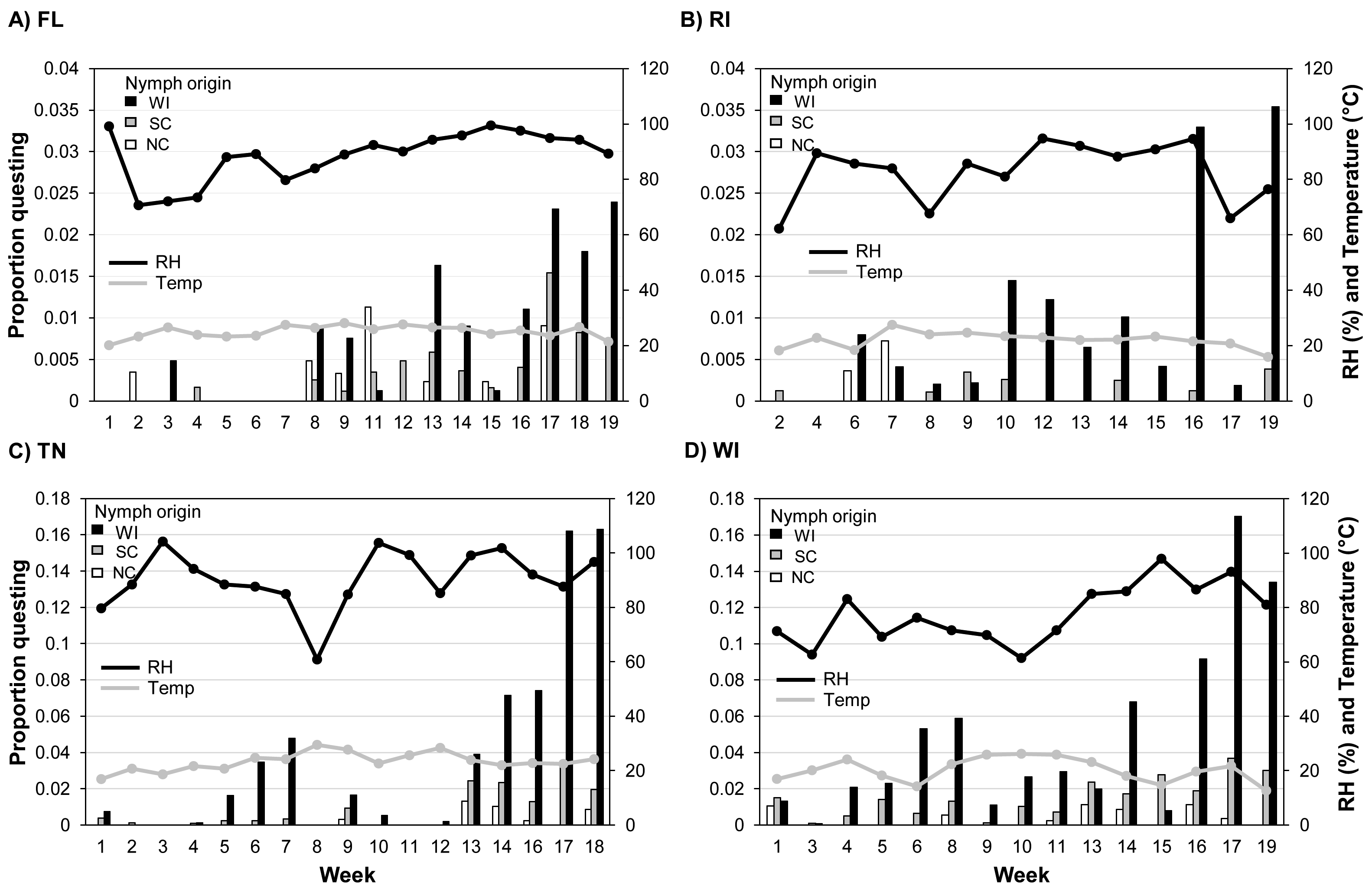

Supplement: S1 Fig — Mean proportion of questing nymphs (by nymph origin-WI, SC, NC) observed in arenas during weekly observations (bar graphs, primary y-axis) at the 4 field sites in 2012: (A) Florida, (B) Rhode Island, (C) Tennessee and (D) Wisconsin. Mean ambient (10 cm) temperature and relative humidity readings for each observation week are expressed by line graphs with values on the secondary y-axis. The first column (panels A and C) shows data for the southern, non-endemic sites (FL and TN), the second column (panel B and D) shows data for the northern, endemic sites (RI and WI). NOTE: Primary y-axis differs for top and bottom rows. Although we did observe increased activity for all three nymph origins during the second half of the 2012 observation period, on average, WI nymphs (black bars) quested at higher proportions than SC and NC nymphs (grey and white bars) throughout the entire observation period and at all sites. The data used in this figure are given in S6 Data. (TIF) [file pone.0127450.s009.tif]

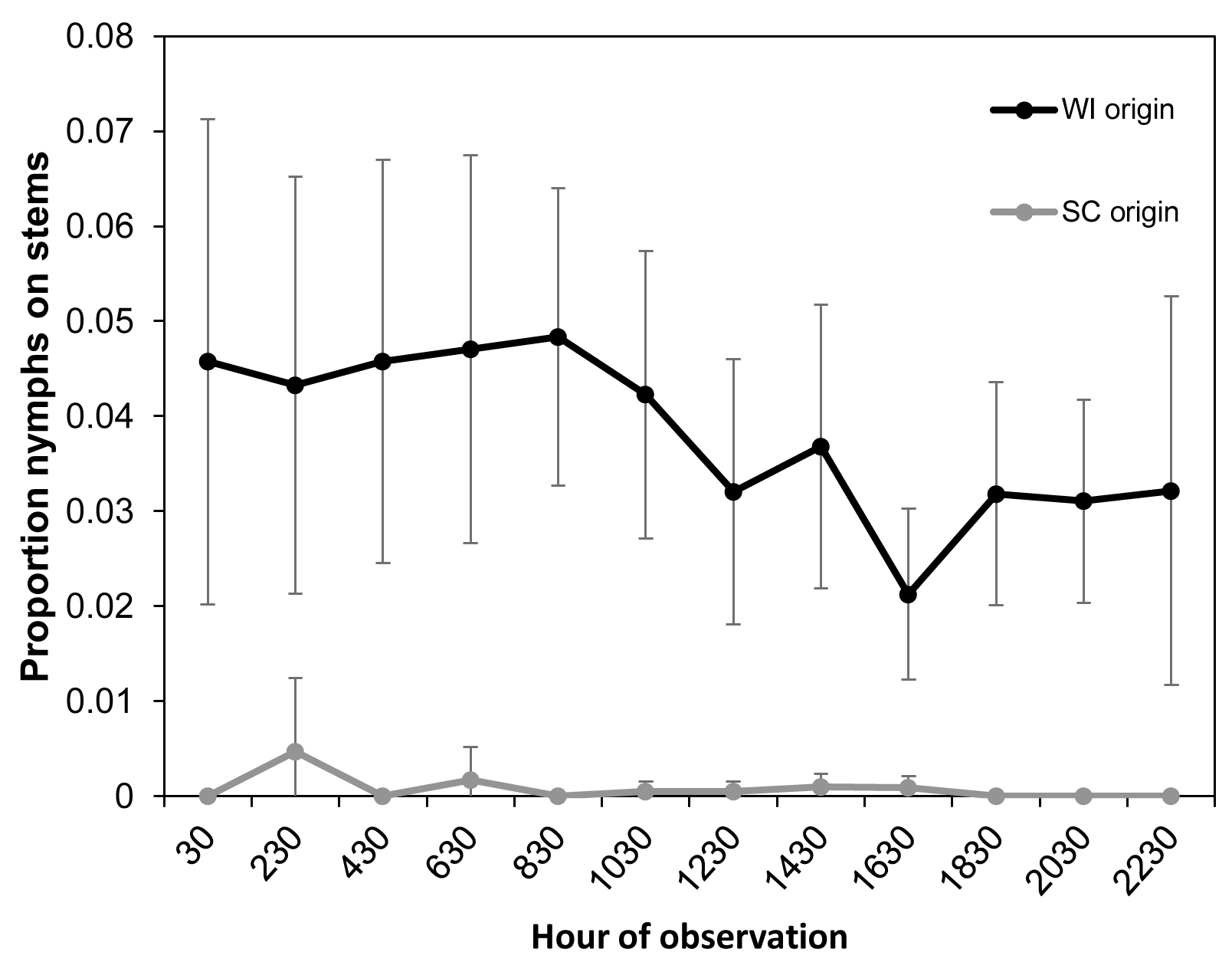

Supplement: S2 Fig — Proportion of nymphal I. scapularis (means ± 95CIs) of northern (WI) and southern (SC) U.S. origin, questing on stems during each observation time, in outdoor arenas in Wisconsin, June-July 2011. For both groups, emergence was highest before 0830 hours and dropped steadily with the exception of a small peak observed in the late afternoon (1230–1630 hours). The data used in this figure are given in S7 Data. (TIF) [file pone.0127450.s010.tif]

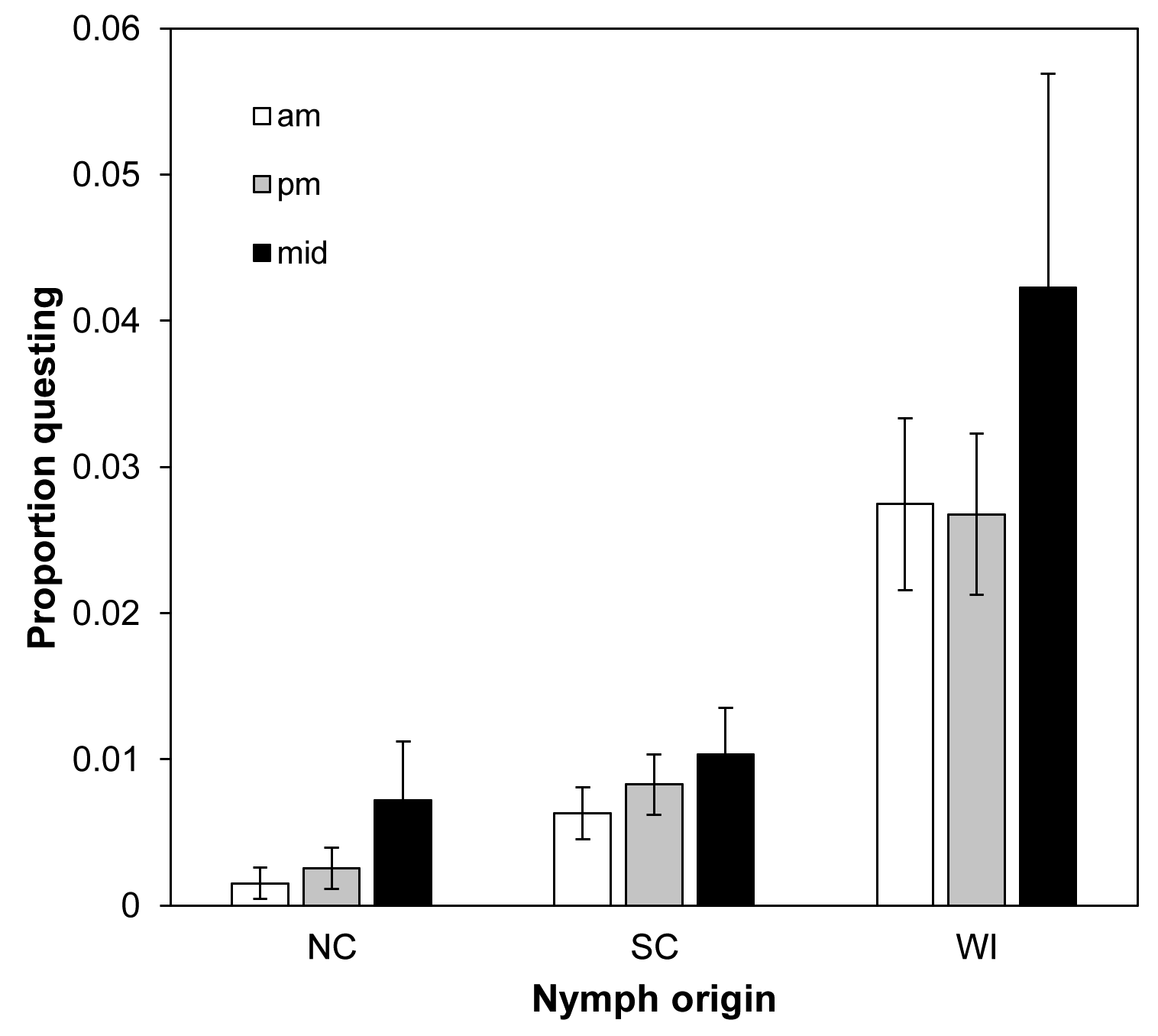

Supplement: S3 Fig — Proportion of nymphal I. scapularis (means ± 95 CIs) of northern (WI) and southern (NC, SC) origin, observed questing at all four sites during each observation hour (am = ~0800 hours, pm = ~1600 hours, mid = ~0000 hours), in outdoor arenas at 4 sites (WI, RI, TN, FL) in 2012. Questing was highest during the midnight observations; am and pm observations yielded similar numbers of ticks for all 3 origins. The data used in this figure are given in S8 Data. (TIF) [file pone.0127450.s011.tif]

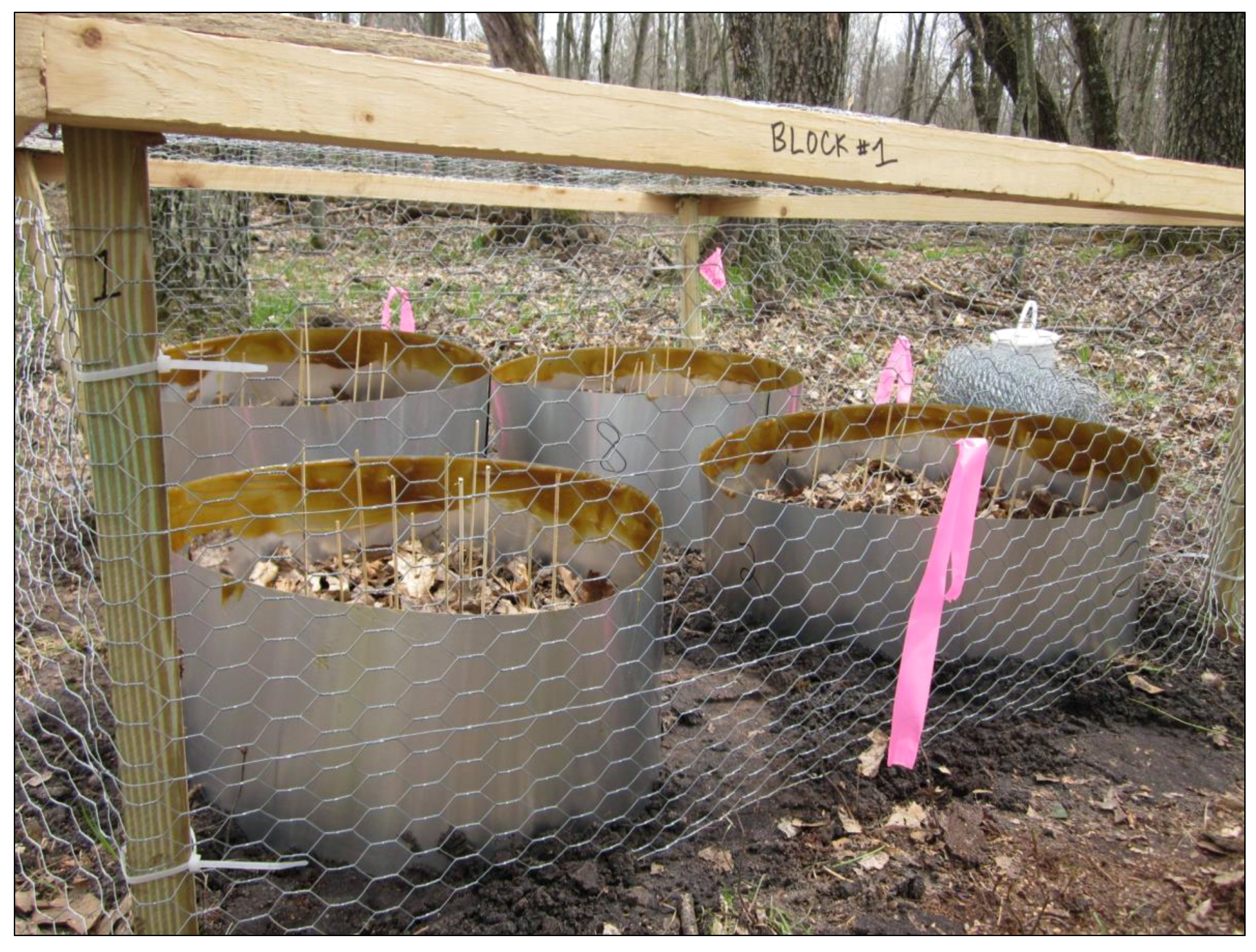

Supplement: S4 Fig — Arenas were grouped in blocks of four and surrounded with wire mesh. (TIF) [file pone.0127450.s012.tif]
