# Supplementary material for: Different Populations of Blacklegged Tick Nymphs Exhibit Differences in Questing Behavior That Have Implications for Human Lyme Disease Risk
Source: PLoS One. 2015 May 21;10(5):e0127450. doi: 10.1371/journal.pone.0127450 (PMC4440738; doi:10.1371/journal.pone.0127450)
Supplement: S1 Table — Posterior mean difference in predicted probability of questing between origins for each site where nymphs were observed. The asterisks and bolded font indicates those comparisons for which a credible difference (HDIs do not include zero) has been determined. The data shown in this table are given in S1 Data (2011) and S3 Data (2012), and the R code that generated it is found in S1 Text. (DOCX) [file pone.0127450.s013.docx]

| **Year** | **Site where tested** | **Posterior probability of difference in questing means (95% HDIs)** | | |
| --- | --- | --- | --- | --- |
|  |  | **WI - SC** | **WI - NC** | **SC - NC** |
| **2011** | WI | **0.032 (0.004,0.076)*** | - | - |
|  |  |  |  |  |
| **2012** | FL | **0.004**  **(7e-5, 0.009)*** | **0.005**  **(6e-4, 0.010)*** | 0.001  (-0.001, 0.003) |
|  | TN | **0.015**  **(0.002, 0.031)*** | **0.018**  **(0.005, 0.036)*** | 0.003  (-1e-4, 0.008) |
|  | RI | **0.003**  **(0.001, 0.006)*** | **0.003**  **(0.001, 0.007)*** | 4e-4  (-5e-5, 0.001) |
|  | WI | **0.015**  **(0.002, 0.034)*** | **0.018**  **(0.004, 0.036)*** | 0.003  (-2e-4, 0.007) |
