# Supplementary material for: Different Populations of Blacklegged Tick Nymphs Exhibit Differences in Questing Behavior That Have Implications for Human Lyme Disease Risk
Source: PLoS One. 2015 May 21;10(5):e0127450. doi: 10.1371/journal.pone.0127450 (PMC4440738; doi:10.1371/journal.pone.0127450)
Supplement: S2 Table — Posterior mean difference in predicted probability of recovery between origins for each site where nymphs were observed. The asterisks and bolded font indicates those comparisons for which a credible difference (HDIs do not include zero) has been determined. The data shown in this table are given in S2 Data (2011) and S4 Data (2012), and the R code that generated it is found in S1 Text. (DOCX) [file pone.0127450.s014.docx]

| **Year** | **Site where tested** | **Posterior probability of difference in recovery means (95% HDIs)** | | | |  |
| --- | --- | --- | --- | --- | --- | --- |
|  |  | **WI - SC** | **WI - NC** | | **SC - NC** | |
| **2011** | WI | -0.026  (-0.118, 0.080) | - | - | |  |
|  |  |  |  |  | |  |
| **2012** | FL | 0.0535  (-0.185, 0.294) | **0.3158**  **(0.104, 0.544)*** | **0.2623**  **(0.038, 0.508)*** | |  |
|  | TN | 0.058  (-0.198, 0.281) | 0.191  (-0.137, 0.452) | 0.133  (-0.160, 0.457) | |  |
|  | RI | 0.193  (-0.009, 0.386) | 0.172  (-0.078, 0.406) | -0.022  (-0.279, 0.193) | |  |
|  | WI | **0.195**  **(0.001, 0.391)*** | 0.209  (-0.027, 0.433) | 0.014  (-0.243, 0.214) | |  |
