# Supplementary material for: Different Populations of Blacklegged Tick Nymphs Exhibit Differences in Questing Behavior That Have Implications for Human Lyme Disease Risk
Source: PLoS One. 2015 May 21;10(5):e0127450. doi: 10.1371/journal.pone.0127450 (PMC4440738; doi:10.1371/journal.pone.0127450)
Supplement: S3 Table — Temperature and relative humidity (means ± SD) at leaf litter (level “0 cm”) or above leaf litter (level “10 cm” = ambient) inside arenas at each field site in 2011 and 2012. Fort McCoy, Wisconsin was the only study site in 2011. In 2012, readings were discontinued after the second week of September. Latitude coordinates obtained from nationalatlas.gov. The data shown in this table are given in S5 Data. (DOCX) [file pone.0127450.s015.docx]

| **Field site** | **Latitude (°N)** | **Months sampled** | **Temp (°C) 0cm** | **Temp(°C) 10cm** | **Relative humidity (%) 0cm** | **Relative humidity (%) 10cm** |
| --- | --- | --- | --- | --- | --- | --- |
| Fort McCoy, WI | 44.04 | 2011: June-July | 20.1 | 21.4 | 93.4 | 85.1 |
|  |  |  | (5.1 | (6.7) | (11.5) | (19.3) |
|  |  | 2012: May-Sep. | 17.9 | 19.5 | 101.5 | 78.8 |
|  |  |  | (4.2) | (6.9) | (5.8) | (19.2) |
|  |  |  |  |  |  |  |
| Kingston, RI | 41.48 | 2012: May-Sep. | 18.8  (4.4) | 19.5  (5.1) | 94.9  (10.8) | 90.8  (15.4) |
|  |  |  |  |  |  |  |
| Oak Ridge, TN | 36.01 | 2012: May-Sep. | 21.7  (3.9) | 22.4  (4.3) | 96.6  (7.7) | 93.5  (12.6) |
|  |  |  |  |  |  |  |
| Tall Timbers Research Station, FL | 30.53 | 2012: May-Sep. | 23.3  (2.5) | 24.3  (3.5) | 97.0  (9.5) | 91.5  (13.7) |
|  |  |  |  |  |  |  |
