# Supplementary material for: Different Populations of Blacklegged Tick Nymphs Exhibit Differences in Questing Behavior That Have Implications for Human Lyme Disease Risk
Source: PLoS One. 2015 May 21;10(5):e0127450. doi: 10.1371/journal.pone.0127450 (PMC4440738; doi:10.1371/journal.pone.0127450)
Supplement: S4 Table — Number of clutches (mothers) used to propagate nymphs for behavior experiments in 2011 and 2012. Engorged females were collected from hunter harvested deer in fall of 2010 in Wisconsin (WI2010) and South Carolina (SC2010) and produced nymphs for 2011 experiments. Nymphs for the 2012 experiments were offspring of the nymphal cohort raised from the females collected in 2010. Two of 2012 clutches (WIF2,2010*) were directly related to the WI clutches used in 2011 arenas, while the remaining 5 clutches (WIF2,2010, SCF2,2010) were derived from mothers collected at the same time (but not related to) as those who provided clutches for 2011 arenas. Additional engorged females were collected from deer in North Carolina (NC2011) and South Carolina (SC2011) in fall of 2011 and were used to supplement the 2012 nymph supply. A single arena always contained nymphs from the same geographic origin (WI, SC or NC), however nymphs within an arena could have all been siblings from a single clutch (homogeneous) or a mixture of siblings and non-siblings from multiple clutches (heterogeneous). (DOCX) [file pone.0127450.s016.docx]

| **Year** | **Engorged female origin** | **# clutches**  **propagated** | | **Composition of arenas** | | **Number of arenas containing nymphs of a given origin (per site)** | | | |
| --- | --- | --- | --- | --- | --- | --- | --- | --- | --- |
|  |  |  |  | **Homogeneous** | **Heterogeneous** | **WI** | **RI** | **TN** | **FL** |
| **2011** | WI_2010_ | | 8 | 7 | 1 | 8 | - | - | - |
|  | SC_2010_ | | 14 | 4 | 4 | 8 | - | - | - |
|  |  | |  | **Total number of arenas** | | **16** |  |  |  |
| **2012** | WI_F2,2010*_ | 2 | | 7 | 1 | 2 | 2 | 2 | 2 |
|  | WI_F2,2010_ | 1 | | 1 | 11 | 3 | 3 | 3 | 3 |
|  | SC_F2,2010_ | 4 | | 12 | 15 | 8 | 7 | 6 | 6 |
|  | SC_2011_ | 2 | | 7 | 0 | 2 | 1 | 2 | 2 |
|  | NC_2011_ | 3 | | 12 | 0 | 3 | 3 | 3 | 3 |
|  |  |  | | **Total number of arenas** | | **18** | **16** | **16** | **16** |
|  |  |  | |  | |  |  |  |  |
