# Supplementary material for: Different Populations of Blacklegged Tick Nymphs Exhibit Differences in Questing Behavior That Have Implications for Human Lyme Disease Risk
Source: PLoS One. 2015 May 21;10(5):e0127450. doi: 10.1371/journal.pone.0127450 (PMC4440738; doi:10.1371/journal.pone.0127450)
Supplement: S1 Text — (DOCX) [file pone.0127450.s017.docx]

**Annotated R-code used to generate Table 1, Table 2, S1 Table, and S2 Table**

*#load 'rethinking' package (Map2stan)*

library(rethinking)

**########## Analysis: Nymph Questing 2011 (Year 1) #####################**

*# Year 1: Ticks from 2 origins (WI & SC) observed at 1 site (WI)*

*# Sampling scheme: 4 dates ("samples") on which bi-hourly observations were conducted on the arenas #for 24 hours periods (13 total observations per sample, except sample 2 was only 14 hours long).*

*#load dataset for 2011 questing data*

data.2011<-read.csv("E:\\ S1 Data.csv")

table(data.2011$nn.dowels)

*# Make WI origin the reference group, create dummy variable for SC origin*

data.2011$originSC <- ifelse( data.2011$nn.origin=="SC" , 1 , 0 )

data.2011$arena_id <- as.integer( as.factor( data.2011$arena ) )

data.2011$hour_id <- as.integer( as.factor( data.2011$hour) )

data.2011$sample_id <- as.integer( as.factor( data.2011$sample ) )

*# Fit model 1, 2011: Log-odds nymphs on dowels. Random effects: arena, hour, sample; Random slopes: # hour, sample*

m1.2011 <- map2stan(

alist(

nn.dowels ~ dbinom(nn.released,p),

logit(p) <- a + a_arena + a_hour + a_sample +

(bSC + bSCh + bSCs)*originSC,

a ~ dnorm (0,10),

bSC ~ dnorm(0,10),

a_arena[arena_id] ~ dnorm(0,sigma_arena),

c(a_hour,bSCh)[hour_id] ~ dmvnorm2(0,sigma_hour,Rho_hour),

c(a_sample,bSCs)[sample_id] ~ dmvnorm2(0,sigma_sample,Rho_sample),

sigma_arena ~ dcauchy(0,1),

sigma_hour ~ dcauchy(0,1),

sigma_sample ~ dcauchy(0,1),

Rho_hour ~ lkj_corr(4),

Rho_sample ~ lkj_corr(4)

),

data=data.2011,

start=list(

a=0, bSC=0,

a_arena=rep(0,16),

a_hour=rep(0,12),

bSCh=rep(0,12),

a_sample=rep(0,4),

bSCs=rep(0,4),

sigma_arena=1,

sigma_hour=rep(1,2),

sigma_sample=rep(1,2),

Rho_hour=diag(2),

Rho_sample=diag(2)

),

warmup=1000,iter=3000 , sample=TRUE

)

*# check convergence - trace plots*

plot(m1.2011)

*# extract samples from posterior and use for calculations*

post.2011 <- extract.samples(m1.2011)

*# compute posterior probability quest for each origin (WI & SC), for average arena, hour, and sample*

dowels.WI <- logistic(post.2011$a) #(WI emergence)

dowels.SC <-logistic(post.2011$a + post.2011$bSC) #(SC emergence)

*#* ***Table 1****: posterior probability nymphs on dowels 2011*

mean(dowels.WI)

HPDI(dowels.WI)

mean(dowels.SC)

HPDI(dowels.SC)

*#* ***Table 1****: effect size 2011*

mean(dowels.WI)/mean(dowels.SC)

*#* ***S1 Table****: posterior probability origin difference 2011 (WI-SC difference)*

mean(dowels.WI-dowels.SC)

HPDI(dowels.WI-dowels.SC)

**############## Analysis: Nymph recovery (survival) 2011 ################**

*# load dataset for 2011 survival*

data.2011.surv <-read.csv("E:\\ S2 Data.csv")

*# Make WI origin the reference group, create dummy variable for SC origin*

data.2011.surv$originSC <- ifelse( data.2011.surv$nn_origin=="SC" , 1 , 0 )

data.2011.surv$arena_id <- as.integer( as.factor( data.2011.surv$arena_field ) )

*# Fit model 2, 2011: Log-odds recovery of nymphs from arenas in 2011. Random effect on arena*

m2.2011 <- map2stan(

alist(

nn_recovered ~ dbinom(nn_released,p),

logit(p) <- a + a_arena+

bSC*originSC,

a ~ dnorm(0,10),

a_arena[arena_id] ~ dnorm(0,sigma_arena),

bSC ~ dnorm(0,10)),

data=data.2011.surv,

start=list(

a=0, bSC=0,

a_arena=rep(0,16),

sigma_arena=1),

warmup=1000,iter=3000 , sample=TRUE)

*# check convergence - trace plots*

plot(m2.2011)

*# extract samples from the posterior*

post.2011surv<-extract.samples(m2.2011)

*# compute posterior probability recovery for each origin, for average arena, hour, and sample*

surv2011.WI <- logistic(post.2011surv$a) #(WI survival)

surv2011.SC <-logistic(post.2011surv$a + post.2011surv$bSC) #(SC survival)

*#* ***Table 2****: Posterior probability recovery for 2011*

mean(surv2011.WI)

HPDI(surv2011.WI)

mean(surv2011.SC)

HPDI(surv2011.SC)

*#* ***Table 2****: effect size recovery for 2011*

mean(surv2011.WI)/mean(surv2011.SC)

*#* ***S2 Table****: posterior probability origin difference 2011 recovery (WI-SC difference)*

mean(surv2011.WI-surv2011.SC)

HPDI(surv2011.WI-surv2011.SC)

**############## Analysis: Nymph Questing 2012 (Year 2) ##############**

*#Year 2: Ticks from 3 origins (WI, SC, NC) observed at 4 sites (FL, RI, TN, WI)*

*# Sampling scheme: Weekly/bi-weekly samples (morning and afternoon = 2 observations/week) #collected simultaneously at 4 sites in the eastern U.S.*

*# load dataset for 2012 questing in tick gardens*

data.2012<-read.csv("E:\\S3 Data.csv")

table(data.2012$nn.total)

*# Make WI origin the reference group, create dummy variable for SC & NC origins*

data.2012$originSC <- ifelse( data.2012$nn.origin=="SC" , 1 , 0 )

data.2012$originNC <- ifelse( data.2012$nn.origin=="NC" , 1 , 0 )

data.2012$arena_id <- as.integer( as.factor( data.2012$arena ) )

data.2012$site_id <- as.integer( as.factor( data.2012$site ) )

data.2012$week_id <- as.integer( as.factor( data.2012$week ) )

data.2012$week.s <- ( data.2012$week - mean(data.2012$week) ) / sd( data.2012$week )

*# Fit model 1, 2012: Log-odds nymphs emerged from leaf litter. Random effects: arena, site, week; # Random slopes: week, site*

m1.2012 <- map2stan(

alist(

nn.total ~ dbinom(nn.released,p),

logit(p) <- a + a_arena + a_site + a_week +

(bSC + bSCw + bSCs)*originSC +

(bNC + bNCw + bNCs)*originNC,

a ~ dnorm(0,10),

bSC ~ dnorm(0,10),

bNC ~ dnorm(0,10),

a_arena[arena_id] ~ dnorm(0,sigma_arena), # varying intercepts for arena

c(a_site,bSCs,bNCs)[site_id] ~ dmvnorm2(0,sigma_site,Rho_site), # varying intercept & slope for site

c(a_week,bSCw,bNCw)[week_id] ~ dmvnorm2(0,sigma_week,Rho_week), # varying inercept & slope for week

sigma_arena ~ dcauchy(0,1),

sigma_site ~ dcauchy(0,1),

sigma_week ~ dcauchy(0,1),

Rho_week ~ lkj_corr(4),

Rho_site ~ lkj_corr(4)

),

data=data.2012,

start=list(

a=0,bSC=0,bNC=0,

a_arena=rep(0,66),

a_site=rep(0,4),

bSCs=rep(0,4),

bNCs=rep(0,4),

a_week=rep(0,19),

bSCw=rep(0,19),

bNCw=rep(0,19),

sigma_arena=1,

sigma_site=rep(1,3),

sigma_week=rep(1,3),

Rho_week=diag(3),

Rho_site=diag(3)

),

warmup=1000,iter=3000 , sample=TRUE

)

# check convergence - trace plots

plot(m1.2012)

*# extract samples from the posterior*

post.2012 <- extract.samples(m1.2012)

*# compute posterior probability quest for each origin, at site(i), for average arena and week*

*#* ***Must recalculate for each site (change site #)***

**# Site 1=FL, 2=RI, 3=TN, 4=WI**

p.link <- function( originSC=0 , originNC=0 , site=1 ) {

p <- with( post.2012 ,

logistic(

a + a_site[,site] +

(bSC + bSCs[,site])*originSC +

(bNC + bNCs[,site])*originNC

)

)

return(p)

}

use_site <- 1

p.WI <- p.link( 0 , 0 , use_site )

p.SC <- p.link( 1 , 0 , use_site )

p.NC <- p.link( 0 , 1 , use_site )

*#* ***Table 1****: posterior probability emergence 2012*

mean(p.WI)

HPDI( p.WI , 0.95 )

mean(p.SC)

HPDI( p.SC , 0.95 )

mean(p.NC)

HPDI( p.NC , 0.95 )

*#* ***Table 1****: effect size (ratio of emergence 2012)*

mean(p.WI)/mean(p.SC)

mean(p.WI)/mean(p.NC)

mean(p.SC)/mean(p.NC)

*#* ***S1 Table****: posterior probability of differences among origins*

mean(p.WI-p.SC)

HPDI(p.WI-p.SC)

mean(p.WI-p.NC)

HPDI(p.WI-p.NC)

mean(p.SC-p.NC)

HPDI(p.SC-p.NC)

**############## Analysis: Nymph recovery 2012 ################**

*# load dataset for 2012 survival (recovery)*

data.2012.surv <-read.csv("E:\\ S4 Data.csv")

*# Make WI origin the reference group, create dummy variable for SC & NC origins*

data.2012.surv$originSC <- ifelse( data.2012.surv$nn_origin=="SC" , 1 , 0 )

data.2012.surv$originNC <- ifelse( data.2012.surv$nn_origin=="NC" , 1 , 0 )

data.2012.surv$arena_id <- as.integer( as.factor( data.2012.surv$arena ) )

data.2012.surv$site_id <- as.integer( as.factor( data.2012.surv$site ) )

*# Fit model 2, 2012: Log-odds recovery of nymphs from arenas in 2012. Random effects: arena, site; # Random slope: site*

m2.2012 <- map2stan(

alist(

nn_recovered ~ dbinom(nn_released,p),

logit(p) <- a + a_arena + a_site +

(bSC + bSCs)*originSC +

(bNC + bNCs)*originNC,

a ~ dnorm(0,10),

bSC ~ dnorm(0,10),

bNC ~ dnorm(0,10),

a_arena[arena_id] ~ dnorm(0,sigma_arena), # varying intercepts for arena

c(a_site,bSCs,bNCs)[site_id] ~ dmvnorm2(0,sigma_site,Rho_site), # varying intercept & slope for site

sigma_arena ~ dcauchy(0,1),

sigma_site ~ dcauchy(0,1),

Rho_site ~ lkj_corr(4)

),

data=data.2012.surv,

start=list(

a=0,bSC=0,bNC=0,

a_arena=rep(0,66),

a_site=rep(0,4),

bSCs=rep(0,4),

bNCs=rep(0,4),

sigma_arena=1,

sigma_site=rep(1,3),

Rho_site=diag(3)

),

warmup=1000,iter=3000 , sample=TRUE

)

*# check convergence - trace plots*

plot(m2.2012)

*# extract samples from the posterior*

post.2012surv <- extract.samples(m2.2012)

*# compute posterior probability of recovery for each origin, at site(i), for average arena*

***# Must recalculate for each site*** *(change site #)*

***# Site 1=FL, 2=RI, 3=TN, 4=WI***

p.link <- function( originSC=0 , originNC=0 , site=1 ) {

p <- with( post.2012surv ,

logistic(

a + a_site[,site] +

(bSC + bSCs[,site])*originSC +

(bNC + bNCs[,site])*originNC

)

)

return(p)

}

use_site <- 1

surv2012.WI <- p.link( 0 , 0 , use_site )

surv2012.SC <- p.link( 1 , 0 , use_site )

surv2012.NC <- p.link( 0 , 1 , use_site )

*#* ***Table 2****: posterior probability recovery 2012*

mean(surv2012.WI)

HPDI(surv2012.WI)

mean(surv2012.SC)

HPDI(surv2012.SC)

mean(surv2012.NC)

HPDI(surv2012.NC)

*#* ***Table 2:*** *effect size (ratio of recovery 2012)*

mean(surv2012.WI)/mean(surv2012.SC)

mean(surv2012.WI)/mean(surv2012.NC)

mean(surv2012.SC)/mean(surv2012.NC)

*#* ***S2 Tabl****e: posterior probability origin difference 2012 recovery*

mean(surv2012.WI-surv2012.SC)

HPDI(surv2012.WI-surv2012.SC)

mean(surv2012.WI-surv2012.NC)

HPDI(surv2012.WI-surv2012.NC)

mean(surv2012.SC-surv2012.NC)

HPDI(surv2012.SC-surv2012.NC)
